# Sea level rise and fall north of Greenland reorganize Arctic freshwater export to North Atlantic

**DOI:** 10.1038/s41467-026-75610-8

**Published:** 2026-07-15

**Authors:** Qiang Wang, Qi Shu, Caili Liu, Shizhu Wang, Sergey Danilov, Jiao Chen, Thomas Jung

**Affiliations:** 1 Alfred Wegener Institute, Helmholtz Center for Polar and Marine Research, Bremerhaven, Germany; 2 First Institute of Oceanography and Key Laboratory of Marine Science and Numerical Modeling, Ministry of Natural Resources, Qingdao, China; 3 Institute of Environmental Physics, University of Bremen, Bremen, Germany

**Keywords:** Physical oceanography, Physical oceanography

## Abstract

Arctic freshwater is exported to the North Atlantic through Fram Strait and the Canadian Arctic Archipelago, and its partitioning influences subpolar ocean stratification. How climate warming will alter these export pathways remains unclear. Here we show that dynamic sea level north of Greenland initially rises as the Beaufort Gyre freshens, but falls again as saltier Eurasian waters intrude once global warming exceeds approximately 3 K above preindustrial levels. This nonmonotonic sea-level evolution reorients surface geostrophic circulation north of Greenland and reorganizes Arctic liquid freshwater export pathways. As a result, the share of Arctic Ocean volume export through Fram Strait increases by about 40% before declining again. Satellite observations already indicate rising sea levels in the Last Ice Area, suggesting that this transition may be underway and that Fram Strait is emerging as a major Arctic export gateway for liquid freshwater and associated chemical tracers. Such changes could have important implications for North Atlantic salinity, biogeochemistry, and climate.

## Abstract (web summary)

The Fram Strait could become a dominant route for Arctic liquid freshwater export under climate change, with implications for the North Atlantic. Rising sea levels north of Greenland suggest that this transition may already be underway.

## Introduction

The Arctic Ocean exerts a strong influence on regional climate and marine ecosystems through its export of freshwater and chemical tracers to the North Atlantic. It receives freshwater from river runoff, precipitation, and sea ice melt, and exports this freshwater to the subpolar North Atlantic^1,2^. There, it modulates upper-ocean stratification, dense water formation, and the Atlantic meridional overturning circulation (AMOC)^3–5^. These processes, in turn, affect climate over both North America and Eurasia^6^. Arctic rivers also deliver large amounts of terrigenous nutrients and dissolved organic carbon to the Arctic Ocean^7,8^, while Pacific inflow supplies additional nutrients and carbon-rich waters^9^. Once exported into the North Atlantic, Arctic waters enriched in freshwater, nutrients, and dissolved organic carbon can alter biogeochemical processes in downstream regions^10–13^. Under continued warming, permafrost thaw and intensified coastal erosion are releasing stored contaminants into the Arctic Ocean^14^, potentially increasing ecological stress in the North Atlantic.

Arctic freshwater and associated tracers are exported to the subpolar North Atlantic through two gateways: Fram Strait east of Greenland and the Canadian Arctic Archipelago (CAA) west of Greenland^15–18^. The impacts of freshwater on the AMOC are sensitive to its location^19^. Freshwater exported through Fram Strait tends to exert a larger weakening effect on the AMOC because it more effectively increases stratification in convection regions. In contrast, freshwater exported through the CAA can be advected rapidly by the Labrador Current, often passing deep convection sites with relatively limited impact on the overturning circulation^20–22^. Furthermore, the subpolar North Atlantic contains distinct ecosystems on either side of Greenland, shaped by contrasting currents and water masses^23,24^. Consequently, changes in the partitioning of Arctic freshwater and chemical tracers between the two pathways might influence these ecosystems.

Sea surface height (SSH) in both the Arctic coastal region north of the CAA and northern Baffin Bay can influence the variability of volume transport through the CAA^25–27^. Dynamic sea level anomalies originating in the subpolar gyre can propagate rapidly as coastally trapped waves along western Greenland into northern Baffin Bay; elevated (reduced) dynamic sea level there weakens (enhances) the CAA outflow^25,26^. In contrast, dynamic sea level changes north of the CAA affect the transport through the CAA in the opposite sense^25,26^. In addition, shifts in Arctic Ocean circulation regimes can also alter the partitioning of Arctic exports between the two gateways^28–30^.

After accumulating freshwater for more than a decade^31–33^, the Beaufort Gyre began releasing part of this reservoir in the late 2010s^34–36^, raising dynamic sea level north of the CAA^36^. The coastal region extending from the western CAA to northern Greenland is also referred to as the Last Ice Area (LIA) because it is expected to be the last Arctic region to retain multiyear sea ice^37^. Although dynamic sea level in the LIA has increased in recent years^36^, it remains unclear whether this reflects decadal variability or signals an emergence of a long-term trend. Given the importance of export partitioning between the CAA and Fram Strait, it is essential to identify the processes that primarily determine Arctic export partitioning in a warming climate.

However, projections of future ocean change remain highly uncertain, particularly in the Arctic Ocean^38–43^. An important source of uncertainty is persistent bias in simulated Arctic Ocean circulation in CMIP6 models. High-resolution simulations substantially improve the representation of Arctic hydrography and circulation^44^ and are therefore needed to provide more robust projections of dynamic sea level change and Arctic-subarctic fluxes.

In this study, using high-resolution model simulations, we find that dynamic sea level north of Greenland rises until global mean surface temperature reaches  ~ 3 K above preindustrial, and then declines. The associated reorientation of surface geostrophic currents dominates over other processes, first increasing and then decreasing the fraction of Arctic export through Fram Strait, thereby reorganizing the distribution of Arctic-sourced waters in the North Atlantic. The recently observed rise in LIA SSH suggests that Arctic liquid freshwater export may already be transitioning toward the projected Fram Strait-dominated regime.

## Results

### Emerging rise in LIA dynamic sea level and Arctic freshwater export

**Fig. 1:**
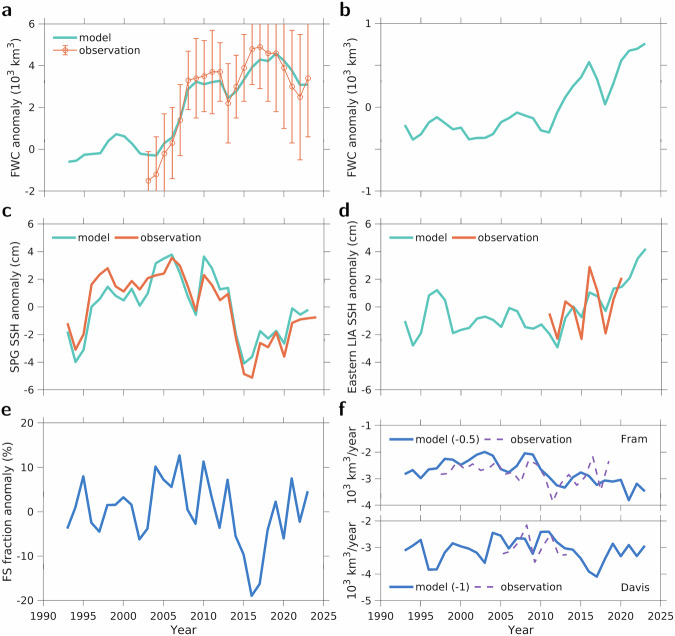
Recent changes in dynamic sea level and Arctic export in observations and hindcast simulation. Anomalies of freshwater content (FWC) in the (**a**) Beaufort Gyre and (**b**) eastern Last Ice Area (LIA). The error bars in (**a**) indicate observational uncertainties reported in the previous study^73^. The location of the eastern LIA is indicated in Fig. 2a. Anomalies of simulated and satellite-observed^47,75^ sea surface height (SSH) in the (**c**) North Atlantic subpolar gyre (SPG) and (**d**) eastern LIA. **e** Anomaly of simulated Fram Strait volume transport fraction relative to the total Arctic export (the sum of Fram Strait and Davis Strait volume transports). **f** Simulated and observed^17,18^ freshwater transports in (upper) Fram and (lower) Davis straits. Model results are shown with shifted values indicated in the legends. Negative values indicate export from the Arctic. Figure created using MATLAB R2022b (The MathWorks Inc., Natick, MA, USA).

**Fig. 2:**
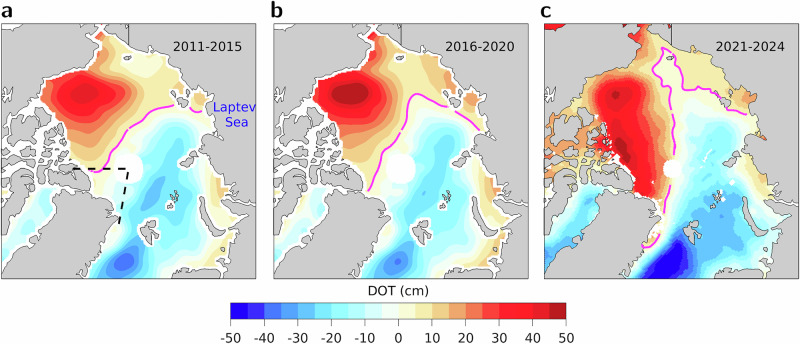
Satellite-observed dynamic ocean topography (DOT) showing changes in ocean circulation. DOT in different periods^47,74^: (**a**) 2011–2015, (**b**) 2016–2020, (**c**) 2021–2024. The anomalies relative to the mean value north of 70^∘^N are shown. The magenta lines indicate the contour lines starting from the outermost eastern Laptev Sea. The panels highlight recent changes in the Beaufort Gyre state, the cyclonic circulation in the Eurasian Arctic, and the orientation of the Transpolar Drift. In (**a**), the black dashed lines mark the eastern Last Ice Area (LIA). Figure created using MATLAB R2022b (The MathWorks Inc., Natick, MA, USA).

The Arctic Beaufort Gyre started accumulating freshwater in the mid-2000s, reaching an unprecedented level in the observational record by the late 2010s (Fig. 1a)^33,35^. Since then, part of this excess freshwater has been released under a cyclonic wind anomaly over the Canada Basin, increasing freshwater content in the eastern LIA (Fig. 1b) and thereby elevating SSH in that region (Fig. 1d and Fig. 2)^36^. These changes are consistently captured by available observations and the hindcast simulation (Fig. 1a–d). The Beaufort Gyre freshwater release has coincided with an intensification of cyclonic circulation in the Eurasian Arctic since the mid-2010s^45^, which further contributed to elevated SSH in the eastern LIA (Fig. 1d and Fig. 2).

SSH in the subpolar gyre decreased from the early 2010s onward (Fig. 1c), leading to a pronounced increase in Davis Strait export^46^ and, consequently, a reduction in the Fram Strait export fraction during the mid-to-late 2010s (Fig. 1e). Since then, the SSH has partially recovered but remains below its early-2000s level (Fig. 1c). Notably, despite the relatively low SSH in the subpolar gyre, the Fram Strait export fraction has rebounded (Fig. 1e). This decoupling suggests that a redirection of upper ocean circulation associated with rising SSH in the eastern LIA has contributed to the recovery of the Fram Strait export fraction. In particular, the orientation of the SSH contour lines indicates that surface geostrophic currents have become more aligned toward the Fram Strait (Fig. 2b, c). Consistent with increased export of low-salinity water from the Beaufort Gyre region, freshwater flux through the Fram Strait has recently reached its highest level of the past few decades (Fig. 1f).

The recent increase in dynamic sea level in the eastern LIA highlights its role in controlling the partitioning of Arctic Ocean export between the two gateways. To assess how dynamic sea level and Arctic Ocean export may evolve in the future, we employ both dedicated high-resolution simulations and CMIP6 models in the following sections.

### Future changes in dynamic sea level and Arctic circulation

**Fig. 3:**
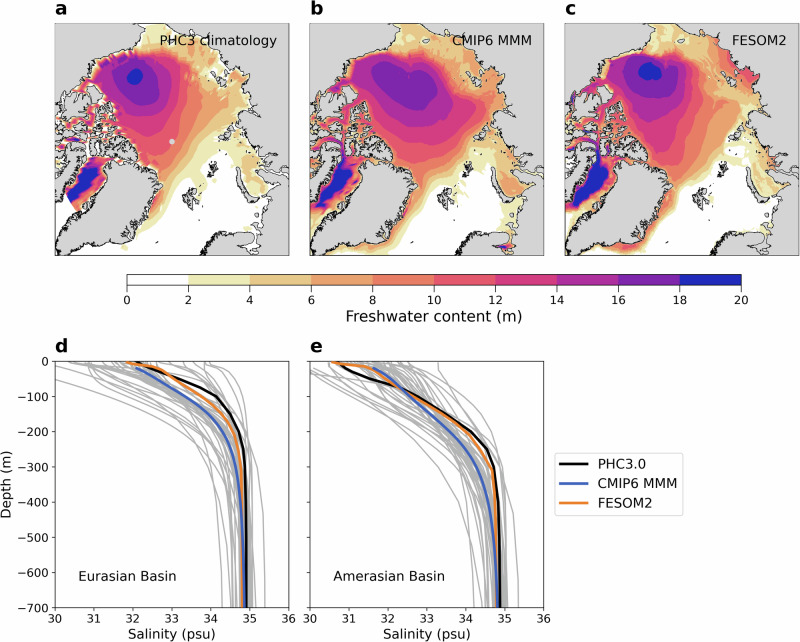
Improved Arctic Ocean representation in high-resolution simulations. Freshwater content (FWC) in (**a**) PHC3 observations^67^, (**b**) CMIP6 multi-model mean (MMM), and (**c**) FESOM2 high-resolution simulations. Vertical profile of salinity averaged in the (**d**) Eurasian Basin and (**e**) Amerasian Basin for PHC3 observations, CMIP6 MMM, and FESOM2 high-resolution simulations. Gray lines indicate individual CMIP6 models. Model results are averaged over the period of 1981–2000.

Because Arctic SSH and the associated surface geostrophic flow are governed primarily by halosteric height (that is, by salinity and freshwater variability)^31,32,47^, numerical models should accurately represent the spatial distribution of Arctic Ocean freshwater. The high-resolution simulation employed here reproduces the observed freshwater distribution reasonably well, including the concentration of the Beaufort Gyre freshwater reservoir to the Canada Basin (Fig. 3a, c). By contrast, CMIP6 models tend to simulate an overly large gyre that nearly occupies the entire Arctic Basin (Fig. 3b). The high-resolution simulation also captures the observed vertical salinity structure, whereas CMIP6 models typically show large biases and substantial inter-model spread (Fig. 3d, e)^38–40^. Furthermore, the high-resolution simulation reasonably represents ocean volume and freshwater transports through Arctic gateways (Supplementary Fig. 1). Given its improved fidelity, this simulation is expected to provide more reliable future projections with reduced uncertainty.

**Fig. 4:**
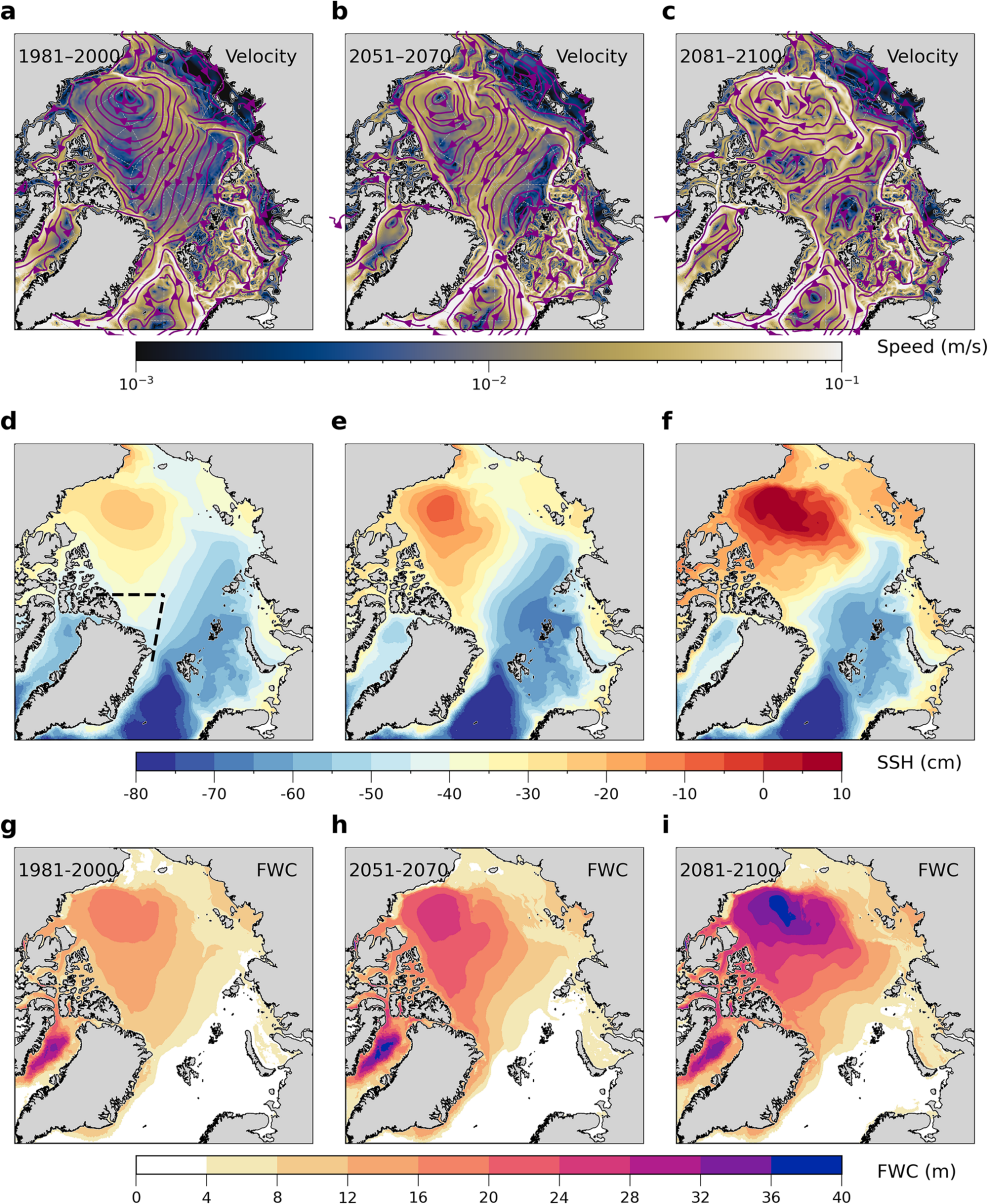
Future evolution of dynamic sea level and circulation in high-resolution model projections. Ocean currents averaged in the upper 100 m and over (**a**) 1981–2000, (**b**) 2051–2070, and (**c**) 2081–2100. Colors show vertically averaged speeds and arrows indicate flow directions. **d**–**f** Same as (**a**–**c**), but for sea surface height (SSH). **g**–**i** Same as (**a**–**c**), but for freshwater content (FWC). In (**d**), the black dashed lines mark the eastern Last Ice Area (LIA).

In this study, we focus mainly on the CMIP6 SSP585 scenario; unless otherwise stated, all projection results refer to this scenario. Projected changes in Arctic freshwater content, SSH and upper-ocean circulation in the high-resolution simulation are shown in Fig. 4. Three periods are considered to characterize these changes: the historical period (1981–2000), the mid-century period (2051–2070), and the late-century period (2081–2100). Surface geostrophic currents derived from SSH closely represent the patterns of ocean circulation averaged over the upper 100 m (cf. Fig. 4a–c and Supplementary Fig. 2), while changes in SSH are mainly determined by changes in freshwater content (cf. Fig. 4d–f and Fig. 4g–i).

**Fig. 5:**
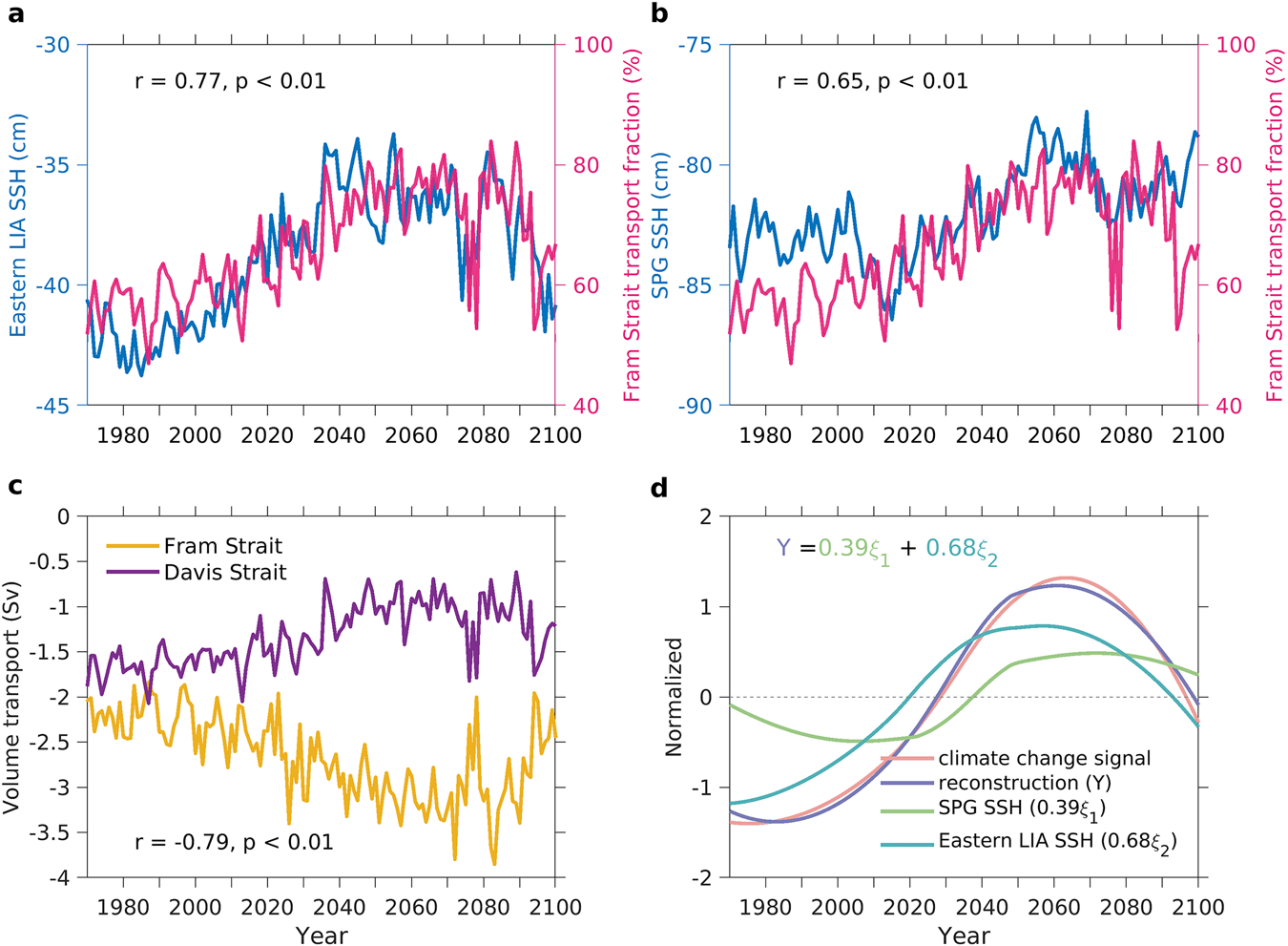
Future changes in Arctic export partitioning mainly determined by sea surface height (SSH) in the eastern Last Ice Area (LIA). **a** SSH in the eastern LIA versus the fraction of Fram Strait volume transport relative to the total Arctic export (the sum of Fram and Davis straits volume transports) in the high-resolution simulation. **b** Same as (**a**), but for SSH in the North Atlantic subpolar gyre (SPG). **c** Volume transports through Fram and Davis straits. Negative values indicate export from the Arctic. **d** Reconstruction of climate change signal of Fram Strait volume transport fraction using SSH in the eastern LIA and in the SPG. The coefficients in the reconstruction indicate that the SSH change in the eastern LIA plays a major role. All the time series are low-pass filtered and normalized before performing multiple regression (see Methods). Figure created using MATLAB R2022b (The MathWorks Inc., Natick, MA, USA).

The model projects a pronounced increase in freshwater content in the Canada Basin (Fig. 4g–i), accompanied by a rise in SSH (Fig. 4d–f) and a strengthening of the anticyclonic Beaufort Gyre circulation (Fig. 4a–c)^44,48^. In addition to Beaufort Gyre freshening, the eastward expansion of freshwater into the eastern LIA leads to increased freshwater content and SSH there by the mid-21st century (Fig. 4e, h and Fig. 5a). However, after the mid-21st century, SSH in the eastern LIA is projected to decrease (Fig. 4f and Fig. 5a).

The anticyclonic Beaufort Gyre and the cyclonic circulation of the Eurasian Arctic are separated by the Transpolar Drift (Fig. 4a)^45^. By the mid-21st century, not only does the Beaufort Gyre strengthen, but the cyclonic circulation in the Eurasian Arctic also intensifies, as indicated by reduced SSH in the Eurasian Basin (Fig. 4d, e). Intensified cyclonic circulation driven by positive Arctic Oscillation forcing is typically associated with an anticlockwise shift of the Transpolar Drift, including an eastward displacement along the Siberian continental slope and a more eastward pathway toward Fram Strait^28,49,50^. Under climate warming, however, the enhanced cyclonic circulation is not accompanied by a clear eastward shift of the Transpolar Drift in the Siberian sector, owing to the opposing influence of the westward expansion of the Beaufort Gyre (Fig. 4b, e). By contrast, near the downstream end of the Transpolar Drift, the pathway shifts toward Fram Strait (Fig. 4b), as both the Beaufort Gyre expansion and the strengthened cyclonic circulation act in concert to increase SSH in the eastern LIA (Fig. 4e).

By the late 21st century, the Beaufort Gyre expands further, leading to a contraction of the cyclonic circulation in the eastern Eurasian Basin (Fig. 4c, f). More saline waters from the Eurasian Basin are advected toward the region north of Greenland (Fig. 4c and Supplementary Fig. 3), increasing salinity (Supplementary Fig. 4a) and thus lowering SSH in the eastern LIA (Fig. 4f). Overall, the eastern LIA is expected to undergo a nonmonotonic evolution in SSH, driven by the combined effects of changes in the Beaufort Gyre and in the cyclonic circulation in the Eurasian Arctic. This evolution strongly influences the orientation of the Transpolar Drift near its downstream end. As shown below, this mechanism is the primary control on the climate-driven redistribution of Arctic export between the two gateways.

### Export partitioning determined by dynamic sea level

The high-resolution simulation reveals a nonmonotonic evolution of volume transport through Fram Strait over the 21st century, increasing until the 2050s and decreasing thereafter (Fig. 5c). Volume transport through Davis Strait exhibits an evolution opposite to that of the Fram Strait (Fig. 5c). The fraction of Fram Strait volume transport relative to total Arctic export (the sum of Fram Strait and Davis Strait transports) is significantly correlated with SSH in the eastern LIA (Fig. 5a). This fraction is also significantly correlated with SSH in the subpolar gyre, although the correlation is weaker (Fig. 5b).

The significant correlations between the Fram Strait export fraction and SSH in the eastern LIA and subpolar gyre suggest that changes in both the Arctic Ocean and the subpolar North Atlantic can influence Arctic export partitioning, consistent with mechanistic understanding from previous studies^25,26^. However, correlations alone do not indicate which factor primarily drives the long-term, climate-forced changes in export partitioning and which factor dominates natural variability. We therefore apply a low-pass filter to isolate the climate-change signal from variability in the Fram Strait export fraction and in SSH in the eastern LIA and the subpolar gyre (Supplementary Fig. 5a–c; see Methods). We then reconstruct the low-pass-filtered Fram Strait export fraction using both low-pass-filtered SSH time series in a multiple linear regression (Fig. 5d). The regression coefficient for SSH in the eastern LIA is approximately 70% larger than that for the subpolar gyre, indicating that the eastern LIA plays a more prominent role in the projected changes in Arctic export partitioning. Consistent with this attribution, the late-21st-century reversal of the export fraction is captured by SSH changes in the eastern LIA, but not by SSH in the subpolar gyre, in the unfiltered time series (Fig. 5a, b).

Fifty-year running correlations between the Fram Strait export fraction and SSH in the eastern LIA and the subpolar gyre for their natural variability components reveals that the relative influence of the two regions shifts over time (Supplementary Fig. 5d–f). During the historical period, SSH variability in the subpolar gyre has a stronger association with Arctic export partitioning, consistent with previous findings that variability in the North Atlantic played a larger role than Arctic changes in driving Davis Strait export variability in the past^51^. After the mid-21st century, SSH variability in the eastern LIA is projected to become increasingly important for that of Arctic export partitioning (Supplementary Fig. 5d–f).

**Fig. 6:**
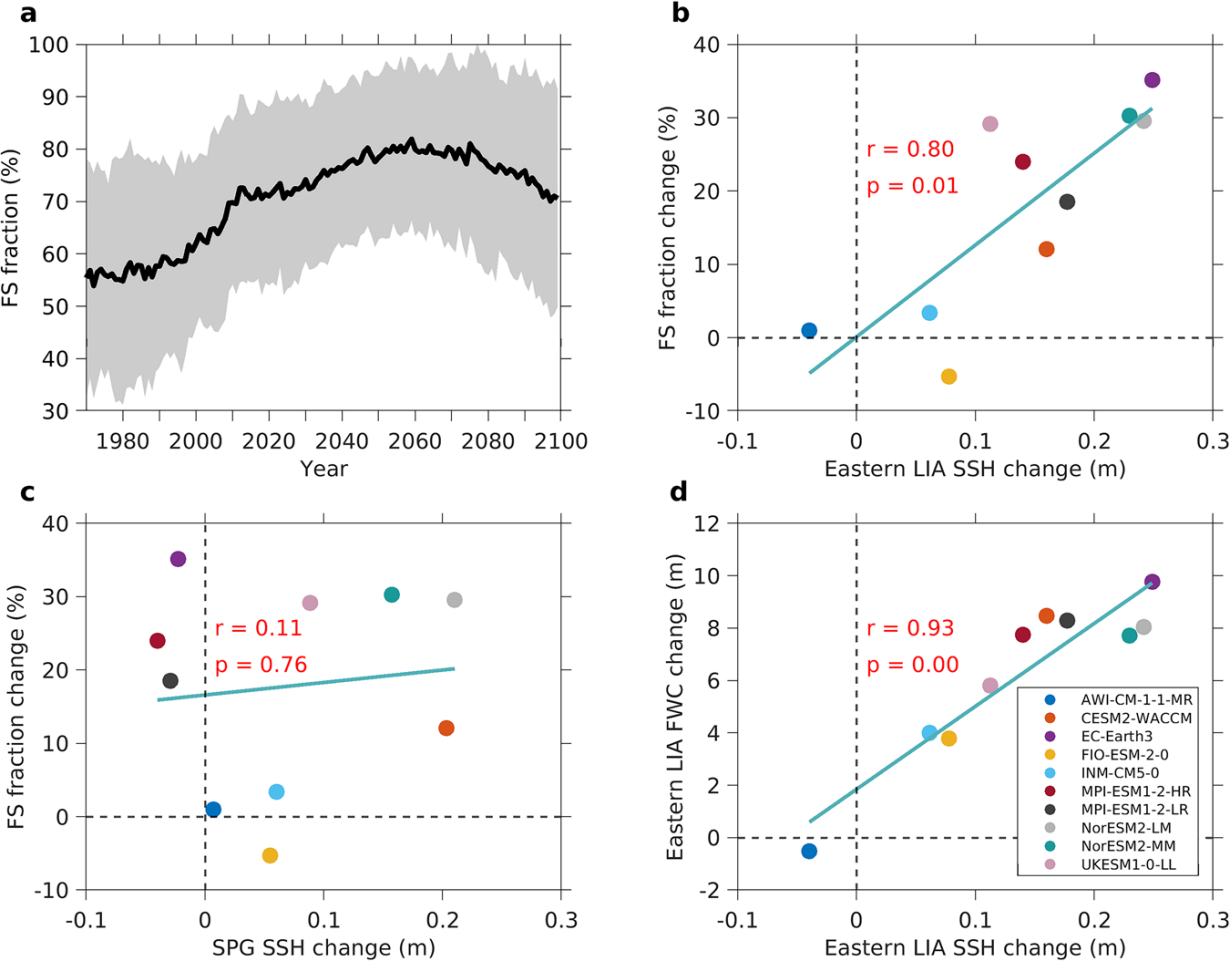
Changes in the partitioning of Arctic exports in CMIP6 models. **a** Fraction of Fram Strait volume transport relative to the total Arctic export (the sum of Fram and Davis straits volume transport). The thick black line shows the multi-model mean (MMM), and the gray shading indicates  ± 1 standard deviation across models. **b** Inter-model correlation between changes in the Fram Strait volume transport fraction and sea surface height (SSH) in the eastern Last Ice Area (LIA). **c** Inter-model correlation between changes in Fram Strait volume transport fraction and SSH in the North Atlantic subpolar gyre (SPG). **d** Inter-model correlation between changes in SSH and freshwater content (FWC) in the eastern LIA. Solid straight lines in (**b-d**) denote linear fits. Temporal changes used in (**b–d**) represent the difference between the periods of 2051–2070 and 1981–2000. Figure created using MATLAB R2022b (The MathWorks Inc., Natick, MA, USA).

Interestingly, the multi-model mean (MMM) of coarse-resolution CMIP6 models also shows a nonmonotonic evolution of the Fram Strait export fraction (Fig. 6a), despite large biases in the simulated positions of the Beaufort Gyre and the Transpolar Drift (Fig. 3b). Owing to these biases, this behavior has previously been regarded as difficult to interpret and possibly unrealistic in CMIP6 assessments^38,40^. Our high-resolution simulation, which provides an improved representation of Arctic Ocean circulation and hydrography, indicates that such nonmonotonic changes in Arctic export through the two gateways are physically plausible. Moreover, the timing and magnitude of the nonmonotonic change in the Fram Strait export fraction in the CMIP6 MMM resemble those obtained in the high-resolution simulation (Fig. 6a). Specifically, the Fram Strait export fraction increases from about 55% in the late 20th century to nearly 80% in the 2050s (Fig. 5a and Fig. 6a), corresponding to an increase of over 40%.

The increase in the Fram Strait export fraction from the historical period to the mid-century period is strongly correlated across CMIP6 models with the increase in SSH in the eastern LIA (Fig. 6b), which is associated with regional freshening (Fig. 6d). By contrast, we find no significant inter-model correlation between changes in the Fram Strait export fraction and SSH in the subpolar gyre (Fig. 6c). These results further underscore the central role of SSH in the eastern LIA in controlling Arctic export partitioning.

Global warming is projected to reach approximately 3 K above the preindustrial level by the mid-21st century under the CMIP6 SSP585 scenario (Supplementary Fig. 6). The analysis above shows that the reversal in the SSH trend in the eastern LIA is projected to occur beyond this threshold (Figs. 5a and 6a). Under the CMIP6 SSP245 scenario, global warming is expected to remain below 3 K during the 21st century (Supplementary Fig. 6). Accordingly, both Fram Strait export and SSH in the eastern LIA are projected to increase without exhibiting a clear reversal (Supplementary Fig. 7). This contrast indicates that the projected trend reversal in Fram Strait export is sensitive to emission pathways and may not occur under moderate warming scenarios. At the same time, the consistent increase in Fram Strait export across scenarios highlights the robustness of the projection that it is likely to become increasingly dominant in the coming decades.

### Dynamical drivers of projected circulation changes

Enhanced air-sea momentum transfer associated with sea ice decline^52^ has been shown to increase Ekman transport from the Eurasian Basin toward the Amerasian Basin, thereby reducing freshwater content in the former and increasing it in the latter^53^. Continued sea ice decline under future warming is therefore expected to further enhance air-sea momentum transfer and promote freshwater accumulation in the Beaufort Gyre^44^. In addition, Arctic near-surface winds are projected to intensify (Supplementary Fig. 8a)^54^, potentially amplifying Arctic Ocean changes.

**Fig. 7:**
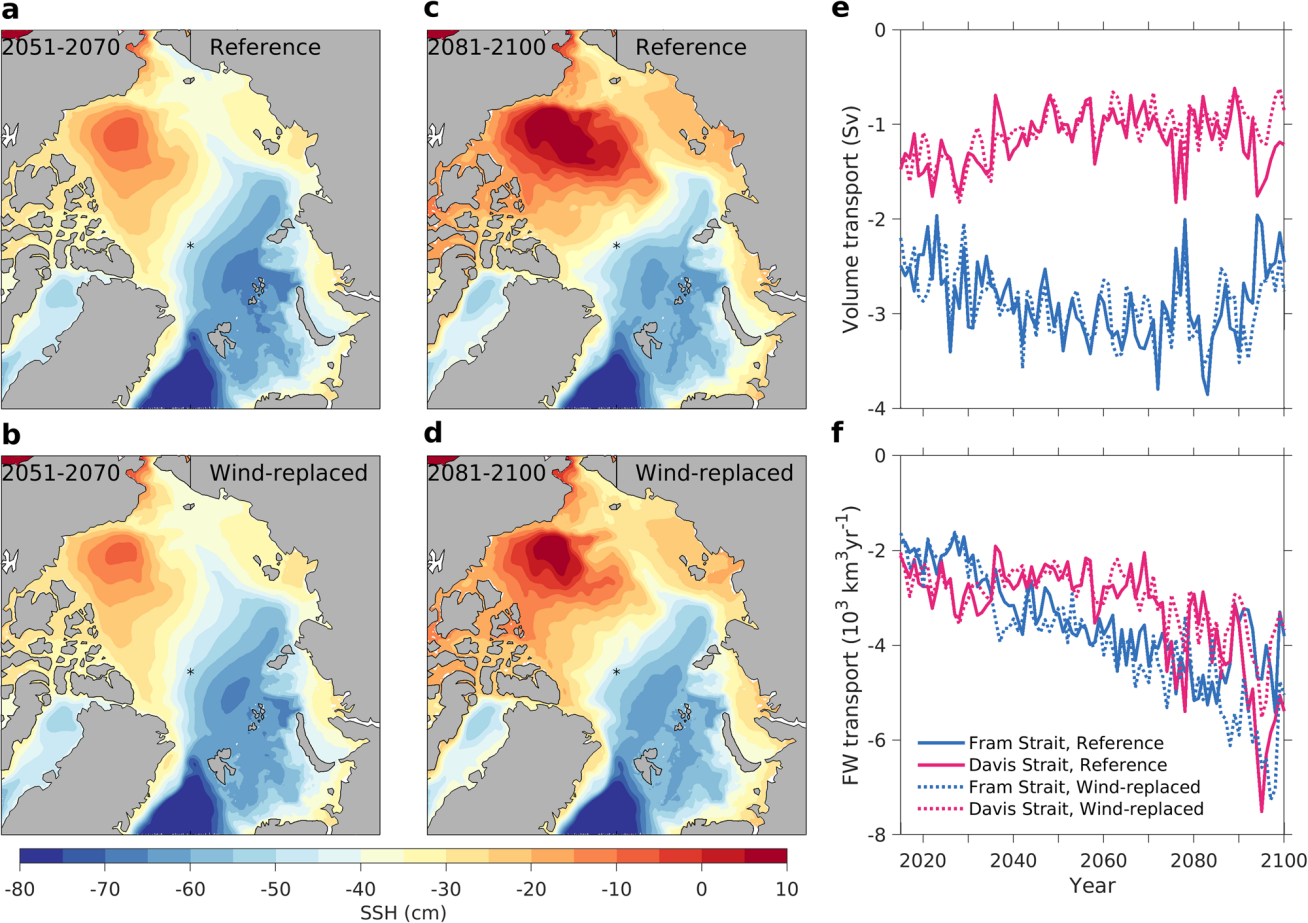
Dynamical drivers of Arctic Ocean circulation and export. **a** Sea surface height (SSH) averaged over 2051–2070 in the reference simulation, that is, the high-resolution future projection. **b** SSH averaged over 2051–2070 in the wind-replaced simulation, in which Arctic winds are replaced by the 20th-century winds. **c**, **d** Same as (**a**, **b**), but for the SSH averaged over 2081–2100. **e** Time series of volume transports in Fram and Davis straits in the two simulations. **f** Same as (**e**), but for freshwater transports in the two gateways. Negative values indicate export from the Arctic. Figure created using MATLAB R2022b (The MathWorks Inc., Natick, MA, USA).

To identify which dynamic factor primarily drives the projected changes in Arctic Ocean circulation, we performed an additional sensitivity simulation in which Arctic near-surface winds in the 21st century were replaced by those from the 20th century (wind-replaced simulation). This wind replacement does not affect the declining trends in summer and winter Arctic sea ice extent (Supplementary Fig. 8b). Up to the mid-21st century, wind intensification exerts only a limited impact on the Beaufort Gyre and the cyclonic circulation in the Eurasian Arctic, as indicated by the similarity in the mid-century SSH between the reference projection and the wind-replaced simulation (Fig. 7a, b). Consequently, SSH in the eastern LIA (Fig. 7a,b), and thus the climate-driven evolution of the volume and freshwater export through the two gateways (Fig. 7e, f), remain largely unchanged.

However, in the late-century period, intensified winds play an increasingly important role in shaping both the anticyclonic and cyclonic circulations (Fig. 7c,d), as only a small seasonal sea ice cover remains (Supplementary Fig. 8b), allowing changes in winds to exert a relatively stronger influence. In the absence of wind intensification, the intrusion of saltier Eurasian waters into the eastern LIA is reduced (Supplementary Fig. 4b, c), weakening the reversal in trends of volume and freshwater export (Fig. 7e, f). These results indicate that sea ice decline drives changes in Arctic Ocean circulation and export throughout the century, whereas the impact of wind strengthening becomes prominent after Arctic sea ice cover is substantially reduced.

### Implications of Arctic water reorganization

**Fig. 8:**
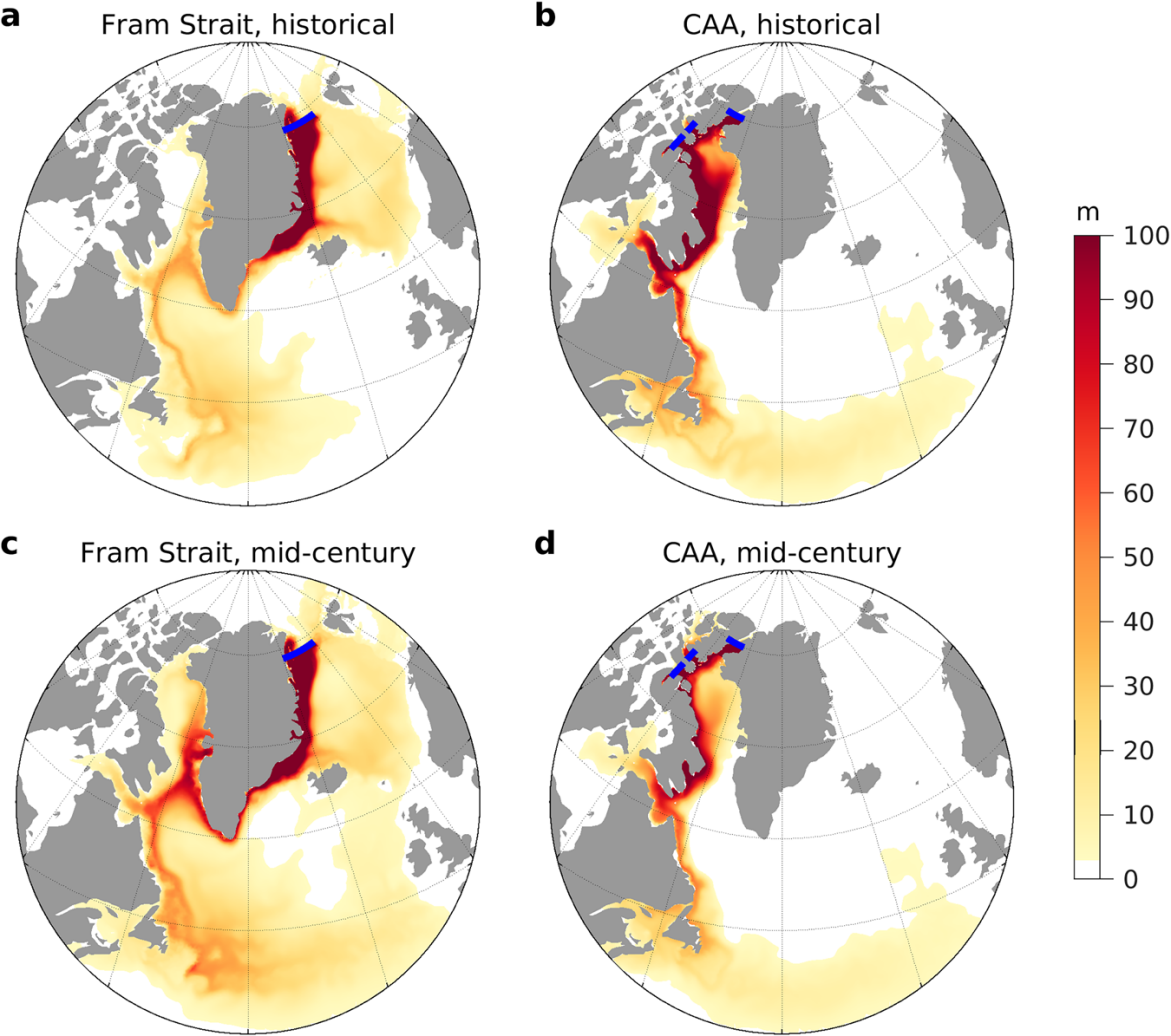
Reorganization of Arctic-sourced waters in the subpolar North Atlantic. **a** Vertically integrated dye tracer concentration for a tracer released in the upper 100 m in the western Fram Strait during the historical period. The release begins in 1980, and the values shown represent the average over the fifth year since release. **b** Same as (**a**), but for a tracer released in the Canadian Arctic Archipelago (CAA) channels. **c**, **d** Same as (**a**, **b**), but for the mid-21st century. In this case, the tracer release begins in 2060, and the values shown represent the average over the fifth year since release. Blue bars indicate the tracer release locations. Figure created using MATLAB R2022b (The MathWorks Inc., Natick, MA, USA).

Dye tracers released at the export gateways clearly illustrate the distribution of Arctic-sourced waters in the subpolar North Atlantic (Fig. 8). Tracer concentrations differ between the two release gateways not only on either side of Greenland, but also in the subpolar gyre (Fig. 8a, b). Within five years, tracers from both gateways reach the southern boundary of the gyre; however, tracers from Fram Strait penetrate farther into the Labrador Sea and the gyre interior. By the mid-21st century, enhanced volume export through Fram Strait increases tracer concentration along its pathway over continental shelf on both sides of Greenland and in the subpolar gyre (Fig. 8c). In contrast, because export through the CAA is reduced, tracer concentration along its pathway, following the outer rim of the gyre relative to the Fram Strait tracer, is lower than in the historical period (Fig. 8d).

The distinct fates of Arctic-sourced waters exported through the two gateways are broadly consistent with previous modeling studies^20,21^. By artificially modifying gateway geometry to redistribute Arctic freshwater export, those studies consistently found that diverting more export through Fram Strait, thereby delivering more freshwater to deep convection areas, weakens the overturning circulation. This implies that the rise in eastern LIA SSH, which increases the Fram Strait export fraction, could similarly contribute to the AMOC decline simulated in our model.

The contrasting spatial distributions of the two tracers also imply potential impacts on regional ecosystems, because Arctic waters differ markedly from North Atlantic waters in nutrient content and carbonate chemistry^12,55^. Pacific Water, for instance, is characterized by relatively low salinity and high nutrient concentrations^56,57^. It is further modified during its transit through the Arctic due to biogeochemical processes and mixing with other water masses^55,58^. To track the fate of Pacific Water, we released an additional dye tracer at Bering Strait. In the historical period, Pacific Water is mainly exported through the CAA, and its spreading closely resembles that of the CAA tracer (Supplementary Fig. 9a). By the mid-century, however, its export is increasingly redirected toward the Fram Strait, leading to a spreading pattern that more closely resembles that of the Fram Strait tracer (Supplementary Fig. 9b). These results highlight how shifts in Arctic export pathways can alter the delivery of biogeochemically distinct water masses to the subpolar North Atlantic. The consequences for regional ecosystems warrant dedicated future study.

## Discussion

We identify the eastern LIA north of Greenland as a key region influencing Arctic impacts on North Atlantic salinity and biogeochemistry because its dynamic sea level strongly modulates climate-driven changes in the partitioning of Arctic Ocean exports. Freshwater accumulation in the Beaufort Gyre during the 2000s and 2010s may have delayed the emergence of sea level rise in the eastern LIA. The recent release of Beaufort Gyre freshwater may have altered the evolution of dynamic sea level in the eastern LIA and shifted Arctic export partitioning toward the trajectory projected by climate models, with liquid freshwater export increasingly routed through Fram Strait. Unless climate mitigation efforts fail and global warming exceeds approximately 3 K above preindustrial levels, Fram Strait is expected to remain the primary oceanic export gateway from the Arctic to the North Atlantic.

The Arctic supplies freshwater to the North Atlantic through both liquid freshwater transported by ocean currents and solid freshwater exported as sea ice. Sea ice export occurs primarily through Fram Strait. However, the ongoing decline of Arctic sea ice has led to a substantial reduction in solid freshwater export^59,60^. As a result, liquid freshwater fluxes are becoming the dominant Arctic freshwater source to the subpolar North Atlantic^51^. In this context, the mechanisms driving the reorganization of liquid freshwater export identified in this study are expected to become increasingly important. At the same time, the continued loss of multiyear sea ice in the LIA^37,61^ is making in situ and satellite observations more feasible, thereby enabling improved monitoring of this climatically important region.

## Methods

### High-resolution climate projections

High-resolution simulations using the Finite Volume Sea ice-Ocean Model (FESOM2)^62–64^ were employed in this study. FESOM2 is a global ocean-sea ice model formulated on unstructured grids, which enables variable horizontal resolution. The horizontal resolution is 4.5 km and 24 km in the Arctic Ocean and North Atlantic, respectively. In most other parts of the global ocean, the horizontal resolution is about 1^∘^. The vertical grid spacing is 5 m at the surface and coarsens with depth, with 47 z-levels in total.

The model was forced using surface forcing fields derived from historical and CMIP6-SSP585 scenario simulations of the coupled climate model FIO-ESM v2.1^65^. The FIO-ESM CMIP6 simulations have a relatively good performance in representing the atmospheric fields in comparison with other CMIP6 models^66^. The forcing fields derived from FIO-ESM v2.1 include near-surface winds, air temperature and specific humidity, downward longwave and shortwave radiation, precipitation, and river runoff. The atmospheric forcing has a spatial resolution of about 1^∘^ and a temporal resolution of three hours.

With this forcing dataset, the FESOM2 simulation was integrated from 1900 to 2100. The model reproduces observed Arctic hydrography and freshwater content (PHC3,^67^) reasonably well and shows clear improvements relative to the CMIP6 models (Fig. 3). In particular, CMIP6 models tend to simulate an overly expansive Beaufort Gyre that occupies most of the Arctic Basin, whereas this bias is substantially reduced in our high-resolution simulation. Furthermore, the simulation reasonably reproduces ocean volume and freshwater transports through Arctic gateways estimated from observations (Supplementary Fig. 1). The 4.5 km horizontal resolution can adequately resolve the two major CAA channels (Parry Channel, about 50 km wide at its narrowest point; Nares Strait, about 30 km wide at its narrowest point), but not the smaller straits (Hell Gate and Cardigan Strait, with a total width of about 10 km)^26^. Overall, the simulated total transports through the CAA as measured at Davis Strait are consistent with the observational estimates and largely fall within the uncertainty range (Supplementary Fig. 1). A detailed evaluation of this simulation was provided along with model data description^68^. The improved fidelity compared to coarse climate models offers a more robust basis for predicting and interpreting future changes in Arctic Ocean circulation.

To demonstrate changes in the spatial distribution of Arctic-sourced water in the North Atlantic, we introduced two passive dye tracers released in the upper 100 m of the Arctic outflows in Fram Strait and straits of the CAA, respectively (release locations indicated in Fig. 8). The tracers were simulated for 1980–1984 and 2060–2064, representing the historical period and mid-century period conditions, respectively. Each tracer was initialized to zero at the start of its simulation window and restored to unity in its corresponding export gateway during the model integration.

To illustrate the impacts of changes in Arctic export partitioning on biogeochemically relevant matter in the subpolar North Atlantic, we introduced a dye tracer restored to unity at Bering Strait to track the fate of Pacific Water. The tracer was released for 14 years starting in 1980 and 2050, respectively. In combination with the Fram Strait and CAA tracers described above, these experiments illustrate how climate change is expected to reorganize the distribution of Pacific-origin water in the subpolar North Atlantic.

The simulation described above is considered as the reference run. We performed an additional simulation for 2015–2100, called wind-replaced simulation. It is identical to the reference simulation except that the wind forcing over the Arctic (defined by the Bering Strait, Barents Sea Opening, Fram Strait, and the northern boundary of the CAA) is replaced with winds from the 20th century. Specifically, in year N of the wind-replaced simulation, the Arctic wind forcing is taken from year N-100. Two dynamic mechanisms have been proposed to be able to affect freshwater accumulation and hence upper ocean circulation in the Arctic Ocean, including enhanced air-sea momentum transfer associated with sea ice decline and the strengthening of Arctic winds^44,54,69^. By construction, the wind-replaced simulation removes the long-term strengthening trend in Arctic winds (Supplementary Fig. 8a). Comparing the reference and wind-replaced simulations therefore allows us to assess the relative importance of these mechanisms.

In this study, we primarily focus on the CMIP6 SSP585 scenario, including the high-resolution simulations described above and the coarse-resolution CMIP6 models described below. SSP585 represents a high-emission pathway characterized by continued fossil fuel-driven development^70^, leading to pronounced warming by the end of the 21st century (Supplementary Fig. 6). To assess the robustness of our main findings and their sensitivity to different warming pathways, we also employed a high-resolution FESOM2 simulation under the SSP245 scenario. SSP245 represents an intermediate pathway with moderate mitigation efforts, resulting in lower global warming compared to SSP585 (Supplementary Fig. 6). This FESOM2 simulation is the same as the reference simulation described above, except for the forcing scenario. All future projection results presented in this paper are based on the SSP585 scenario, except for Supplementary Fig. 7, which shows results from the SSP245 simulation.

We note that, to reduce computational cost and enable multiple century-long simulations, a 4.5 km resolution was applied only in the pan-Arctic region, including the CAA. The resolution in the North Atlantic is insufficient to explicitly resolve mesoscale eddies; therefore, eddy parameterizations were applied to represent their effects on tracer transport and mixing. The SSH variability in the subpolar gyre is well reproduced in comparison with satellite observations (Fig. 1c).

One limitation of the high-resolution simulations used in this study is the absence of air-sea feedback. However, the experimental design is adequate for our main objective to predict and understand changes in Arctic Ocean circulation and export under a prescribed, representative climate trajectory (obtained from a coupled CMIP6 simulation). In this context, the sensitivity experiment, in which Arctic winds are replaced while all other forcing fields are kept unchanged, is feasible and helps to isolate the driving mechanisms.

Additionally, freshwater input from increased melting of the Greenland Ice Sheet was not included in our high-resolution simulations, consistent with CMIP6 climate model simulations. We find that the freshening and expansion of the Beaufort Gyre can drive an increase in dynamic sea level in the eastern LIA. While additional freshwater input may modify the magnitude of these changes and influence ocean circulation, it is unlikely to weaken Beaufort Gyre spinup or diminish its role identified here. Consequently, our main findings regarding the rise in dynamic sea level and the redirection of Arctic export toward Fram Strait are likely to remain qualitatively robust. The accumulation of Greenland meltwater in Baffin Bay and the Labrador Sea could raise the local dynamic sea level, further reducing the share of export through the CAA while increasing the proportion exported through Fram Strait. If so, the export shift toward Fram Strait identified in this study may represent a lower-bound estimate. Nevertheless, quantifying the effects of future land meltwater requires dedicated investigation.

### High-resolution hindcast simulation

A high-resolution ocean-sea ice hindcast simulation forced by atmospheric reanalysis was also employed in this study. This simulation was based on FESOM1.4^71^, configured with the same spatial resolution as the climate projection simulations described above, and forced by the JRA55-do atmospheric reanalysis^72^. Previous studies have shown that this configuration reproduces observed changes in the Arctic Ocean and the North Atlantic subpolar gyre reasonably well^36,46,51^.

We use this hindcast, together with available observations, to assess whether the influence of the eastern LIA on the long-term trend in Arctic export partitioning has emerged. Results from the hindcast simulation are consistent with observations, revealing a recent increase in dynamic sea level in the eastern LIA and its influence on Fram Strait freshwater export (Fig. 1). This consistency also provides an observationally grounded analog for assessing the mechanisms underlying future changes.

### CMIP6 dataset

Historical and projection simulations from 24 CMIP6 coupled climate models were used in this study. We considered the SSP585 scenario^70^, consistent with the scenario used in the reference FESOM2 simulation. The first realization of each of these models was used. CMIP6 models exhibit substantial biases and inter-model spread in the mean state of Arctic Ocean hydrography and circulation (Fig. 3a, b, d, e). Nevertheless, they project future changes in Arctic export partitioning that are consistent with our high-resolution simulations, supporting an important dynamical role of SSH changes in the eastern LIA in controlling the long-term evolution of Arctic export partitioning.

The analyzed CMIP6 models include ACCESS-CM2, ACCESS-ESM1-5, AWI-CM-1-1-MR, CESM2-WACCM, CESM2, CMCC-CM2-SR5, CNRM-CM6-1-HR, CNRM-CM6-1, CNRM-ESM2-1, CanESM5-CanOE, CanESM5, EC-Earth3-Veg, EC-Earth3, FIO-ESM-2-0, GFDL-CM4, HadGEM3-GC31-LL, INM-CM5-0, IPSL-CM6A-LR, MPI-ESM1-2-HR, MPI-ESM1-2-LR, MRI-ESM2-0, NorESM2-LM, NorESM2-MM, UKESM1-0-LL (Supplementary Table 1). They are used to calculate gateway transport time series (Fig. 6a). Ten of these models provided sea level data for both the historical and SSP585 scenario periods, and were used to investigate the relationship between Arctic export partitioning, SSH and freshwater content (shown in Fig. 6b-d).

### Observational dataset

To examine changes over recent decades, we used several observational datasets, including Beaufort Gyre freshwater content derived from in situ measurements^73^, dynamic ocean topography from satellite altimetry for the Arctic^47,74^ and for the subpolar North Atlantic^75^, and freshwater transports through Fram Strait and Davis Strait^17,18^. The synthesized ocean volume and freshwater transports through the major Arctic gateways, derived from inverse modeling and observations^76^, were used to evaluate our simulations. The PHC3 hydrographic climatology^67^ was used to initialize the high-resolution simulations and to evaluate the simulated mean state in the historical period.

### Data analysis

We use SSH anomalies (common terminology in the modeling community) and dynamic ocean topography (DOT) anomalies (used in satellite altimetry) as measures of dynamic sea level variability. Removing the global mean from SSH and DOT isolates the dynamic sea level signal. Accordingly, observed dynamic sea level in the subpolar gyre does not show an upward trend during the satellite era (Fig. 1c), because the global-mean rise, driven primarily by thermal expansion and land-ice mass loss, has been removed.

We calculated the freshwater content in a water column with the following equation:

1 $${{{\rm{FWC}}}}=\int_{H}^{{{{\rm{surface}}}}}({S}_{{{{\rm{ref}}}}}-S)/{S}_{{{{\rm{ref}}}}}{{{\rm{d}}}}z,$$

where *S* is salinity and *S*_ref_ is the reference salinity. The integration is along the vertical coordinate *z* from depth *H* to surface. *H* denotes the depth where salinity reaches the reference salinity. The reference salinity is set to 34.8, the mean salinity of the Arctic Ocean^77^. Integrating (1) over an area provides the total freshwater content in volume for the region.

Ocean volume (VT) and freshwater (FWT) transports through a gateway were calculated as the following:

2 $${{{\rm{VT}}}}=\iint {u}_{n}{{{\rm{d}}}}A,$$

3 $${{{\rm{FWT}}}}=\iint {u}_{n}({S}_{ref}-S)/{S}_{ref}{{{\rm{d}}}}A,$$

where *u*_*n*_ is the ocean velocity component normal to the gateway transect, and the integration is performed over the transect area. Northward velocity is positive, so Arctic export transports have a negative sign.

To separate natural variability and climate change signals, we smoothed the time series of Fram Strait export fraction and SSH using locally estimated scatterplot smoothing^78^ as implemented in MATLAB R2023b. This method performs a sequence of weighted least-squares fits to estimate a smooth trend without assuming a fixed function form. We used a smoothing window with a span of 0.8, meaning that 80% of the nearest data points contribute to each local regression, with weights decreasing with distance^78^. Varying the span between 0.7 and 0.9 produces similar smoothed series and does not affect the main conclusion that projected SSH changes in the eastern LIA dominate the future evolution of Arctic export partitioning. The smoothed time series are interpreted as the climate change signal, and the residuals from the original series represent natural variability (Supplementary Fig. 5a–c). The smoothed time series were normalized, and we then applied multiple linear regression to reconstruct the Fram Strait export fraction using SSH in the eastern LIA and the subpolar gyre (Fig. 5d). A larger regression coefficient for the SSH in the eastern LIA indicates a stronger influence on the export fraction.

Regional winds can induce high-frequency variability on daily to annual time scales in volume transports through Arctic gateways^79^, which is beyond the scope of this study. To focus on low-frequency variability, the residual time series were further smoothed using a five-year running mean (Supplementary Fig. 5d–f). We then calculated running correlations between Fram Strait export fraction and each SSH time series using a 50-year sliding window (Supplementary Fig. 5d–f). Statistically significant correlations indicate that SSH plays an important role in modulating variability in the export partitioning. Our results suggest that the subpolar gyre dominates in the historical period, whereas the influence of the eastern LIA strengthens after about 2050.

## Supplementary information

Supplementary Information

Transparent Peer Review file

## Data Availability

The model data used to produce the paper figures are available at 10.5281/zenodo.20051994.
