# Supplementary material for: Sea level rise and fall north of Greenland reorganize Arctic freshwater export to North Atlantic
Source: Nat Commun. 2026 Jul 15;17:6242. doi: 10.1038/s41467-026-75610-8 (PMC13373228; doi:10.1038/s41467-026-75610-8)
Supplement: Supplementary file 1 — Supplementary Information [file 41467_2026_75610_MOESM1_ESM.pdf]

Supplementary Information for

Sea level rise and fall north of Greenland reorganize Arctic freshwater export to North Atlantic

Qiang Wang[1*], Qi Shu[2*], Caili Liu[2], Shizhu Wang[2], Sergey Danilov[1], Jiao Chen[1], Thomas Jung[1,3]

[1*]Alfred Wegener Institute, Helmholtz Center for Polar and Marine Research, Bremerhaven, 27570, Germany.
[2]First Institute of Oceanography and Key Laboratory of Marine Science and Numerical Modeling, Ministry of Natural Resources, Qingdao, 266061, China.
[3]Institute of Environmental Physics, University of Bremen, Bremen, 28359, Germany.

*Corresponding author(s). E-mail(s): qiang.wang@awi.de; shuqi@fio.org.cn;

Content

Supplementary Figures 1-9
Supplementary Table 1

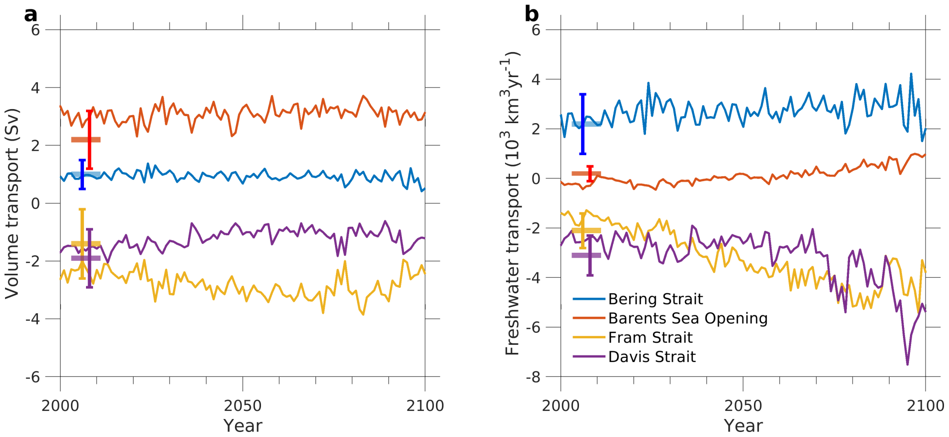

Supplementary Fig. 1: Volume and freshwater transports through Arctic gateways in the high-resolution simulations. (**a**) Volume transports. (**b**) Freshwater transports. The synthesized values obtained by combining observations and inverse modeling [1] are shown as thick lines with ±1 standard deviation. Negative values indicate export from the Arctic. Figure created using MATLAB R2022b (The MathWorks Inc., Natick, MA, USA).

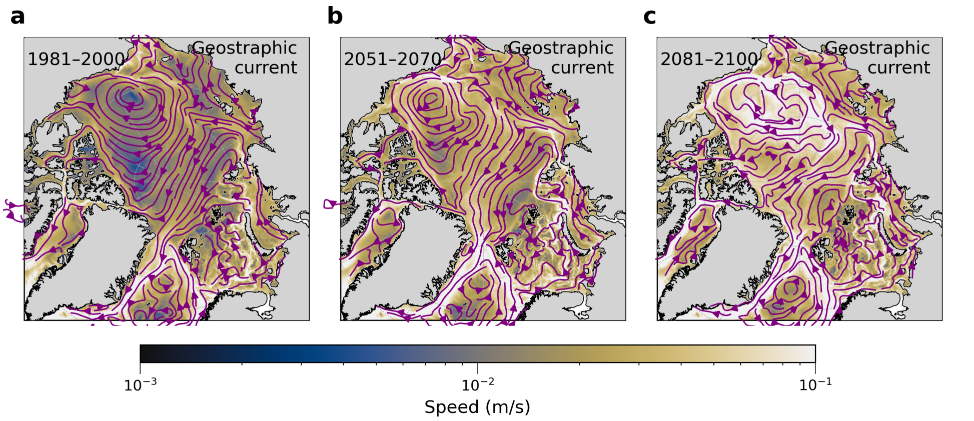

Supplementary Fig. 2: Surface geostrophic currents. (**a-c**) Surface geostrophic currents derived from sea surface height in different periods: (a) 1981–2000, (b) 2051–2070, (c) 2081-2100. Colors show speeds and arrows indicate current directions. The results indicate that changes in the upper ocean circulation shown in Fig. 4a-c are mainly determined by changes in surface geostrophic currents.

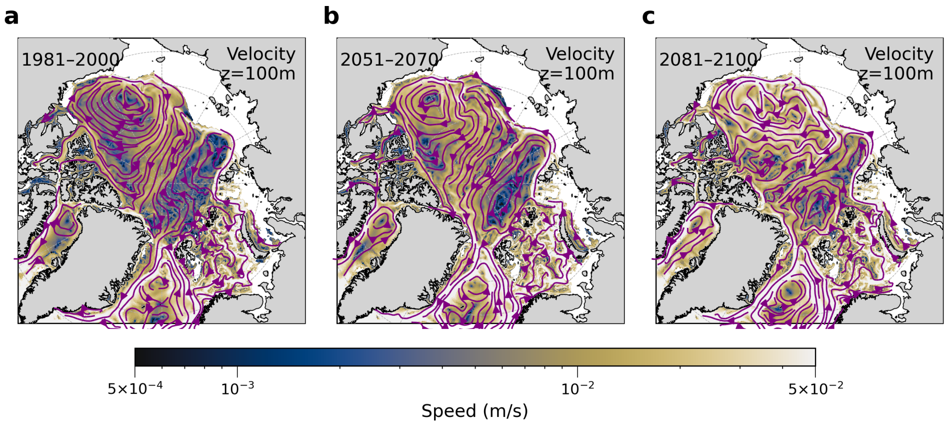

Supplementary Fig. 3: Ocean currents at 100 m depth. (**a-c**) Ocean currents at 100 m depth in different periods: (a) 1981–2000, (b) 2051–2070, (c) 2081-2100. Colors show speeds and arrows indicate current directions. Eurasian waters are advected toward the eastern Last Ice Area north of Greenland in the period of 2081–2100.

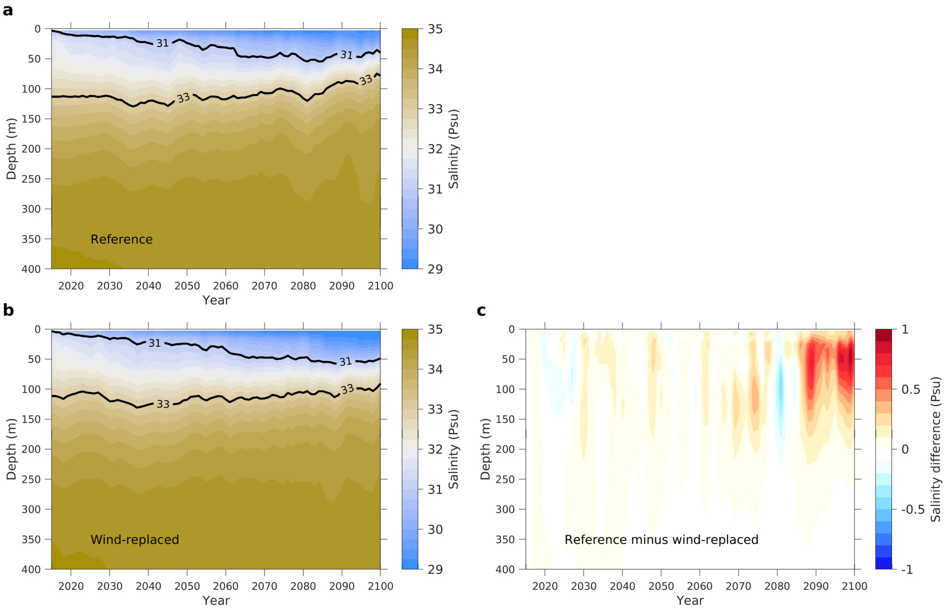

Supplementary Fig. 4: Evolution of salinity averaged over the eastern Last Ice Area (LIA). (**a**) Time-depth plot of salinity in the reference future projection simulations using high-resolution FESOM2. (**b**) Same as (a), but for the wind-replaced simulations, in which Arctic winds are replaced by 20th-century winds. (**c**) Difference between the two simulations. Figure created using MATLAB R2022b (The MathWorks Inc., Natick, MA, USA).

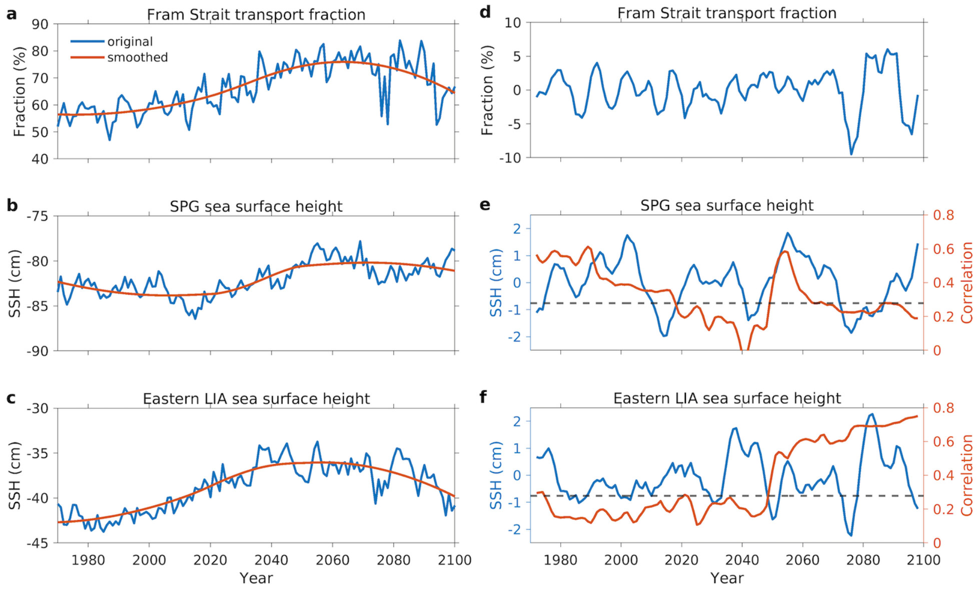

Supplementary Fig. 5: Climate change signals and variability of Arctic export and regional sea surface height (SSH). (**a-c**) Annual-mean (a) Fram Strait volume transport fraction, (b) SSH in the North Atlantic subpolar gyre (SPG), and (c) SSH in the eastern Last Ice Area (LIA). Blue lines indicate the original time series, and red lines show the low-pass filtered time series, interpreted as the climate change signal (see Methods). The Fram Strait volume transport fraction is defined as the ratio of the Fram Strait volume transport to the sum of the volume transports through Fram Strait and Davis Strait. (**d-f**) Variability of (d) Fram Strait volume transport fraction, (e) SSH in the SPG, and (f) SSH in the eastern LIA. Blue lines show the variability component obtained by subtracting the low-pass filtered time series from the original series (i.e., the difference between the blue and red lines in a-c). These residuals are further smoothed using a five-year running mean to address low-frequency variability. Running correlation coefficients between SSH and the Fram Strait volume transport fraction, calculated using a 50-year sliding window, are shown as red lines in (e) and (f). Dashed lines indicate the 95% significance level. Figure created using MATLAB R2022b (The MathWorks Inc., Natick, MA, USA).

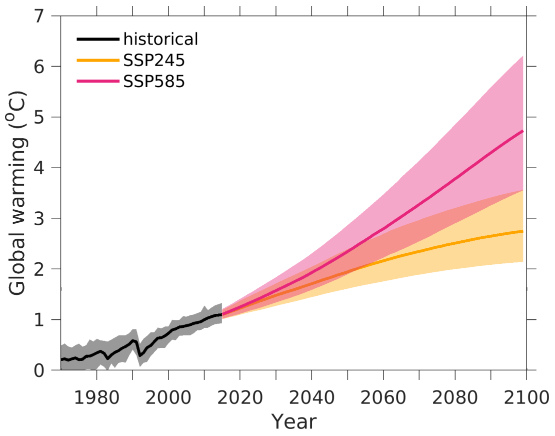

Supplementary Fig. 6: Global warming relative to the pre-industrial level under different scenarios. The solid lines show CMIP6 multi-model mean, and shadings indicate the 5th-95th percentile range [2]. Figure created using MATLAB R2022b (The MathWorks Inc., Natick, MA, USA).

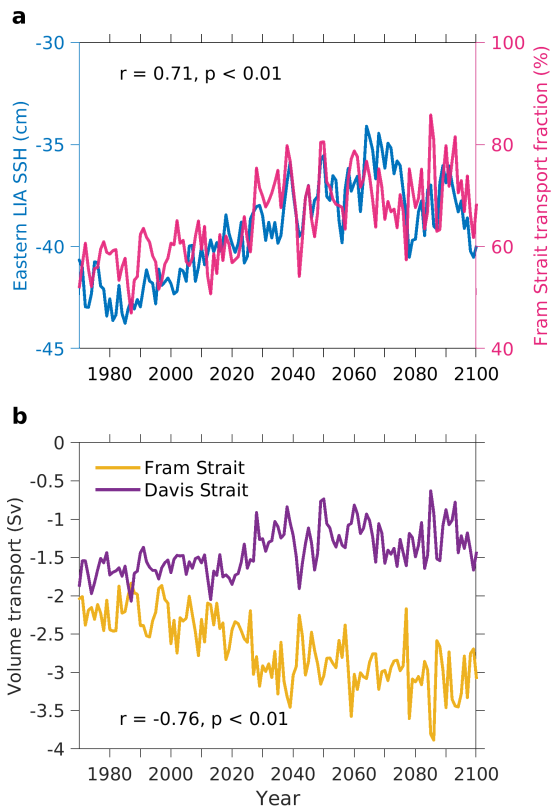

Supplementary Fig. 7: Changes in Arctic exports and sea surface height (SSH) under the SSP245 scenario. (**a**) SSH in the eastern Last Ice Area (LIA) versus the fraction of Fram Strait volume transport relative to total Arctic export (the sum of Fram and Davis straits volume transports) in the high-resolution simulation. (**b**) Volume transports through Fram and Davis straits. Negative values indicate export from the Arctic. Correlation coefficients between the two variables in each panel are shown. Under the SSP245 scenario, the Fram Strait export is projected to increase, without a clear reversal in the 21st century. Figure created using MATLAB R2022b (The MathWorks Inc., Natick, MA, USA).

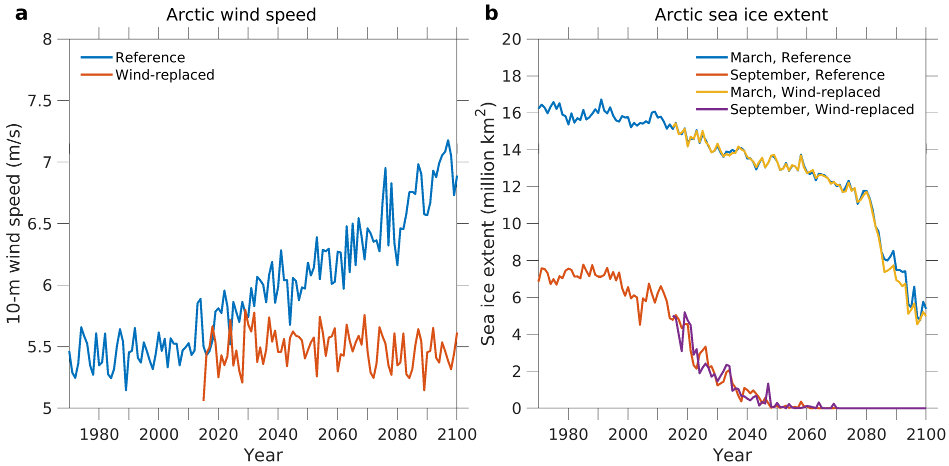

Supplementary Fig. 8: Changes in Arctic wind speeds and sea ice extent. (**a**) Arctic mean wind speeds. The blue line shows the wind forcing used in the reference future projection simulations. The red line indicates the wind forcing used in the wind-replaced sensitivity simulations, which is taken from the 20th century forcing. For example, winds from 1920 are used in the wind-replaced simulations for 2020. (**b**) Arctic sea ice extent in March and September in the reference and wind-replaced simulations.

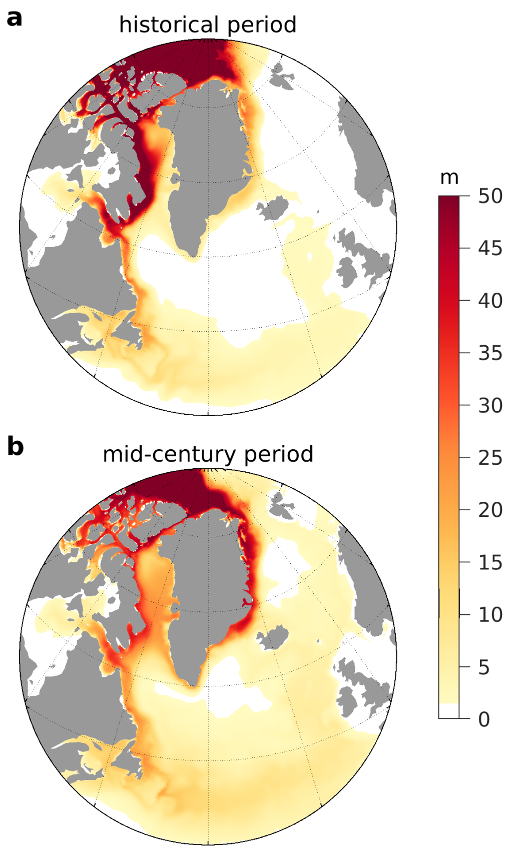

Supplementary Fig. 9: Future changes in the spatial distribution of Pacific Water. (**a,b**) Pacific Water inventory during the (a) historical and (b) mid-century periods. A dye tracer equal to one was release in the Bering Strait starting in 1980 and 2050, respectively. Shown is the vertically integrated tracer concentration, representing Pacific Water inventory, averaged over 1994 and 2064 (that is, 14 years after the start of the release). In the historical period, the spreading of Pacific Water closely resembles that of the Canadian Arctic Archipelago (CAA) tracer shown in Fig. 8b, whereas in the mid-century period it more closely resembles the Fram Strait tracer shown in Fig. 8c. Figure created using MATLAB R2022b (The MathWorks Inc., Natick, MA, USA).

Supplementary Table 1: List of CMIP6 models used in this study. The table includes the number of ocean grid cells (Longitude × Latitude × Depth or number of 2D nodes × Depth) and the references to model data.

No.	Model name	Grid point	Reference
1	ACCESS-CM2	360×300×50	[3, 4]
2	ACCESS-ESM1-5	360×300×50	[5, 6]
3	AWI-CM-1-1-MR	830305×46	[7, 8]
4	CanESM5	360×291×45	[9, 10]
5	CanESM5-CanOE	360×291×45	[11, 12]
6	CESM2	320×384×60	[13, 14]
7	CESM2-WACCM	320×384×60	[15, 16]
8	CMCC-CM2-SR5	362×292×50	[17, 18]
9	CNRM-CM6-1	360×294×75	[19, 20]
10	CNRM-CM6-1-HR	1442×1050×75	[21, 22]
11	CNRM-ESM2-1	360×294×75	[23, 24]
12	EC-Earth3	362×292×75	[25, 26]
13	EC-Earth3-Veg	362×292×75	[27, 28]
14	FIO-ESM-2-0	362×384×60	[29, 30]
15	GFDL-CM4	1440×1080×35	[31, 32]
16	HadGEM3-GC31-LL	360×330×75	[33, 34]
17	INM-CM5-0	360×180×33	[35, 36]
18	IPSL-CM6A-LR	362×332×75	[37, 38]
19	MPI-ESM1-2-HR	802×404×40	[39, 40]
20	MPI-ESM1-2-LR	256×220×40	[41, 42]
21	MRI-ESM2-0	360×363×61	[43, 44]
22	NorESM2-LM	360×385×70	[45, 46]
23	NorESM2-MM	360×385×70	[47, 48]
24	UKESM1-0-LL	360×330×75	[49, 50]

References

[1] Tsubouchi, T., von Appen, W.-J., Kanzow, T. & de Steur, L. Temporal variability of the overturning circulation in the Arctic Ocean and the associated heat and freshwater transports during 2004–10. *Journal of Physical Oceanography* **54**, 81–94 (2024).

[2] Lee, J.-Y. *et al. Future Global Climate: Scenario-Based Projections and Near-Term Information*, 553–672 (Cambridge University Press, Cambridge, United Kingdom and New York, NY, USA, 2021).

[3] Dix, M. *et al.* CSIRO-ARCCSS ACCESS-CM2 model output prepared for CMIP6 CMIP historical (2019). URL https://doi.org/10.22033/ESGF/CMIP6.4271.

[4] Dix, M. *et al.* CSIRO-ARCCSS ACCESS-CM2 model output prepared for CMIP6 ScenarioMIP ssp585 (2019). URL https://doi.org/10.22033/ESGF/CMIP6.4332.

[5] Ziehn, T. *et al.* CSIRO ACCESS-ESM1.5 model output prepared for CMIP6 CMIP historical (2019). URL https://doi.org/10.22033/ESGF/CMIP6.4272.

[6] Ziehn, T. *et al.* CSIRO ACCESS-ESM1.5 model output prepared for CMIP6 ScenarioMIP ssp585 (2019). URL https://doi.org/10.22033/ESGF/CMIP6.4333.

[7] Semmler, T. *et al.* AWI AWI-CM1.1MR model output prepared for CMIP6 CMIP historical (2018). URL https://doi.org/10.22033/ESGF/CMIP6.2686.

[8] Semmler, T. *et al.* AWI AWI-CM1.1MR model output prepared for CMIP6 ScenarioMIP ssp585 (2019). URL https://doi.org/10.22033/ESGF/CMIP6.2817.

[9] Swart, N. C. *et al.* CCCma CanESM5 model output prepared for CMIP6 CMIP historical (2019). URL https://doi.org/10.22033/ESGF/CMIP6.3610.

[10] Swart, N. C. *et al.* CCCma CanESM5 model output prepared for CMIP6 ScenarioMIP ssp585 (2019). URL https://doi.org/10.22033/ESGF/CMIP6.3696.

[11] Swart, N. C. *et al.* CCCma CanESM5-CanOE model output prepared for CMIP6 CMIP historical (2019). URL https://doi.org/10.22033/ESGF/CMIP6.10260.

[12] Swart, N. C. *et al.* CCCma CanESM5-CanOE model output prepared for CMIP6 ScenarioMIP ssp585 (2019). URL https://doi.org/10.22033/ESGF/CMIP6.10276.

[13] Danabasoglu, G. NCAR CESM2 model output prepared for CMIP6 CMIP historical (2019). URL https://doi.org/10.22033/ESGF/CMIP6.7627.

[14] Danabasoglu, G. NCAR CESM2 model output prepared for CMIP6 ScenarioMIP ssp585 (2019). URL https://doi.org/10.22033/ESGF/CMIP6.7768.

[15] Danabasoglu, G. NCAR CESM2-WACCM model output prepared for CMIP6 CMIP historical (2019). URL https://doi.org/10.22033/ESGF/CMIP6.10071.

[16] Danabasoglu, G. NCAR CESM2-WACCM model output prepared for CMIP6 ScenarioMIP ssp585 (2019). URL https://doi.org/10.22033/ESGF/CMIP6.10115.

[17] Lovato, T. & Peano, D. CMCC CMCC-CM2-SR5 model output prepared for CMIP6 CMIP historical (2020). URL https://doi.org/10.22033/ESGF/CMIP6.3825.

[18] Lovato, T. & Peano, D. CMCC CMCC-CM2-SR5 model output prepared for CMIP6 ScenarioMIP ssp585 (2020). URL https://doi.org/10.22033/ESGF/CMIP6.3896.

[19] Voldoire, A. CMIP6 simulations of the CNRM-CERFACS based on CNRM-CM6-1 model for CMIP experiment historical (2018). URL https://doi.org/10.22033/

ESGF/CMIP6.4066.

[20] Voldoire, A. CNRM-CERFACS CNRM-CM6-1 model output prepared for CMIP6 ScenarioMIP ssp585 (2019). URL https://doi.org/10.22033/ESGF/CMIP6.4224.

[21] Voldoire, A. CNRM-CERFACS CNRM-CM6-1-HR model output prepared for CMIP6 CMIP historical (2019). URL https://doi.org/10.22033/ESGF/CMIP6.4067.

[22] Voldoire, A. CNRM-CERFACS CNRM-CM6-1-HR model output prepared for CMIP6 ScenarioMIP ssp585 (2019). URL https://doi.org/10.22033/ESGF/CMIP6.4225.

[23] Seferian, R. CNRM-CERFACS CNRM-ESM2-1 model output prepared for CMIP6 CMIP historical (2018). URL https://doi.org/10.22033/ESGF/CMIP6.4068.

[24] Voldoire, A. CNRM-CERFACS CNRM-ESM2-1 model output prepared for CMIP6 ScenarioMIP ssp585 (2019). URL https://doi.org/10.22033/ESGF/CMIP6.4226.

[25] (EC-Earth), E.-E. C. EC-Earth-Consortium EC-Earth3 model output prepared for CMIP6 CMIP historical (2019). URL https://doi.org/10.22033/ESGF/CMIP6.4700.

[26] (EC-Earth), E.-E. C. EC-Earth-Consortium EC-Earth3 model output prepared for CMIP6 ScenarioMIP ssp585 (2019). URL https://doi.org/10.22033/ESGF/CMIP6.4912.

[27] (EC-Earth), E.-E. C. EC-Earth-Consortium EC-Earth3-Veg model output prepared for CMIP6 CMIP historical (2019). URL https://doi.org/10.22033/ESGF/CMIP6.4706.

[28] (EC-Earth), E.-E. C. EC-Earth-Consortium EC-Earth3-Veg model output prepared for CMIP6 ScenarioMIP ssp585 (2019). URL https://doi.org/10.22033/ESGF/CMIP6.4914.

[29] Song, Z. *et al.* FIO-QLNM FIO-ESM2.0 model output prepared for CMIP6 CMIP historical (2019). URL https://doi.org/10.22033/ESGF/CMIP6.9199.

[30] Song, Z. *et al.* FIO-QLNM FIO-ESM2.0 model output prepared for CMIP6 ScenarioMIP ssp585 (2019). URL https://doi.org/10.22033/ESGF/CMIP6.9214.

[31] Guo, H. *et al.* NOAA-GFDL GFDL-CM4 model output historical (2018). URL https://doi.org/10.22033/ESGF/CMIP6.8594.

[32] Guo, H. *et al.* NOAA-GFDL GFDL-CM4 model output prepared for CMIP6 ScenarioMIP ssp585 (2018). URL https://doi.org/10.22033/ESGF/CMIP6.9268.

[33] Good, P. MOHC HadGEM3-GC31-LL model output prepared for CMIP6 ScenarioMIP ssp585 (2020). URL https://doi.org/10.22033/ESGF/CMIP6.10901.

[34] Ridley, J., Menary, M., Kuhlbrodt, T., Andrews, M. & Andrews, T. MOHC HadGEM3-GC31-LL model output prepared for CMIP6 CMIP historical (2019). URL https://doi.org/10.22033/ESGF/CMIP6.6109.

[35] Volodin, E. *et al.* INM INM-CM5-0 model output prepared for CMIP6 CMIP historical (2019). URL https://doi.org/10.22033/ESGF/CMIP6.5070.

[36] Volodin, E. *et al.* INM INM-CM5-0 model output prepared for CMIP6 ScenarioMIP ssp585 (2019). URL https://doi.org/10.22033/ESGF/CMIP6.12338.

[37] Boucher, O. *et al.* IPSL IPSL-CM6A-LR model output prepared for CMIP6 CMIP historical (2018). URL https://doi.org/10.22033/ESGF/CMIP6.5195.

[38] Boucher, O. *et al.* IPSL IPSL-CM6A-LR model output prepared for CMIP6 ScenarioMIP ssp585 (2019). URL https://doi.org/10.22033/ESGF/CMIP6.5271.

[39] Jungclaus, J. *et al.* MPI-M MPI-ESM1.2-HR model output prepared for CMIP6 CMIP historical (2019). URL https://doi.org/10.22033/ESGF/CMIP6.6594.

[40] Schupfner, M. *et al.* DKRZ MPI-ESM1.2-HR model output prepared for CMIP6 ScenarioMIP ssp585 (2019). URL https://doi.org/10.22033/ESGF/CMIP6.4403.

[41] Wieners, K.-H. *et al.* MPI-M MPI-ESM1.2-LR model output prepared for CMIP6 CMIP historical (2019). URL https://doi.org/10.22033/ESGF/CMIP6.6595.

[42] Wieners, K.-H. *et al.* MPI-M MPI-ESM1.2-LR model output prepared for CMIP6 ScenarioMIP ssp585 (2019). URL https://doi.org/10.22033/ESGF/CMIP6.6705.

[43] Yukimoto, S. *et al.* MRI MRI-ESM2.0 model output prepared for CMIP6 CMIP historical (2019). URL https://doi.org/10.22033/ESGF/CMIP6.6842.

[44] Yukimoto, S. *et al.* MRI MRI-ESM2.0 model output prepared for CMIP6 ScenarioMIP ssp585 (2019). URL https://doi.org/10.22033/ESGF/CMIP6.6929.

[45] Seland, Ø. *et al.* NCC NorESM2-LM model output prepared for CMIP6 CMIP historical (2019). URL https://doi.org/10.22033/ESGF/CMIP6.8036.

[46] Seland, Ø. *et al.* NCC NorESM2-LM model output prepared for CMIP6 ScenarioMIP ssp585 (2019). URL https://doi.org/10.22033/ESGF/CMIP6.8319.

[47] Bentsen, M. *et al.* NCC NorESM2-MM model output prepared for CMIP6 CMIP historical (2019). URL https://doi.org/10.22033/ESGF/CMIP6.8040.

[48] Bentsen, M. *et al.* NCC NorESM2-MM model output prepared for CMIP6 ScenarioMIP ssp585 (2019). URL https://doi.org/10.22033/ESGF/CMIP6.8321.

[49] Good, P. *et al.* MOHC UKESM1.0-LL model output prepared for CMIP6 ScenarioMIP ssp585 (2019). URL https://doi.org/10.22033/ESGF/CMIP6.6405.

[50] Tang, Y. *et al.* MOHC UKESM1.0-LL model output prepared for CMIP6 CMIP historical (2019). URL https://doi.org/10.22033/ESGF/CMIP6.6113.
