# Supplementary material for: Sea level rise and fall north of Greenland reorganize Arctic freshwater export to North Atlantic
Source: Nat Commun. 2026 Jul 15;17:6242. doi: 10.1038/s41467-026-75610-8 (PMC13373228; doi:10.1038/s41467-026-75610-8)
Supplement: Supplementary file 2 — Transparent Peer Review file [file 41467_2026_75610_MOESM2_ESM.pdf]

nature portfolio

Peer Review File

Sea level rise and fall north of Greenland reorganize Arctic freshwater export to North Atlantic

Corresponding Author: Dr Qiang Wang

This file contains all reviewer reports in order by version, followed by all author rebuttals in order by version.

Version 0:

Reviewer comments:

Reviewer #1

(Remarks to the Author)
General Assessment
This manuscript investigates how dynamic sea level changes in the Last Ice Area (LIA) influence the partitioning of Arctic freshwater export between Fram Strait and the Canadian Arctic Archipelago (CAA) using high-resolution ocean–sea ice simulations. The study addresses an important problem for Arctic–North Atlantic coupling and presents an interesting mechanism linking LIA sea level variability with export redistribution.

Overall, the manuscript is clearly written, and the topic is relevant to the Arctic climate community. However, several aspects require clarification or strengthening and I recommend that it be returned for a major revision. In particular:
• The definition of analysis periods and terminology used throughout the manuscript needs clarification.
• The manuscript relies heavily on supplementary figures, including key model evaluation results.
• Several figures require clearer explanations, especially regarding transport sign conventions.
• Some methodological aspects of the model configuration and forcing need further clarification.
• The dynamical mechanism linking sea level changes to gateway transports could be more clearly demonstrated.
• Line numbers appear misaligned throughout the manuscript, which makes it difficult to reference specific parts of the text during review. Please correct this formatting issue.

Below I outline several major and minor comments intended to help improve the clarity and robustness of the manuscript.

Major Comments

1. Manuscript relies heavily on supplementary figures
Several key arguments in the manuscript rely on figures located in the Appendix or Supplementary material. In particular, the evaluation of the model's representation of Arctic freshwater distribution and hydrography is presented primarily in Fig. S1. This comparison is essential because it forms the basis for the authors' claim that the high-resolution simulation realistically reproduces Arctic hydrography and therefore can "provide future projections with reduced uncertainty." However, this evaluation is only briefly described in the text and relies on a figure not included in the main manuscript. I recommend including at least a condensed version of this model validation in the main text (or moving the relevant figure from the Supplementary material), as it is critical for establishing the credibility of the simulations.

2. Representation of transport through the Canadian Arctic Archipelago
The partitioning of Arctic export depends strongly on the representation of flow through the Canadian Arctic Archipelago. The model uses a horizontal resolution of approximately 4.5 km in the Arctic, which may still be marginal for resolving the narrowest CAA channels.
The authors should clarify:
• How well the model grid resolves the major CAA passages.
• Whether transport through the narrowest channels is fully resolved or parameterized.
• Whether any validation of simulated CAA transport against observations has been performed.
Providing additional information on this point would help assess the robustness of the export partitioning results.

3. Resolution asymmetry (Arctic vs North Atlantic)

The model employs high resolution in the Arctic (~4.5 km), but the resolution in the North Atlantic is coarser (~24 km), which does not resolve mesoscale eddies. Since the redistribution of Arctic-sourced waters in the subpolar gyre is influenced by eddy-driven transport, the authors may wish to discuss more explicitly how this limitation could affect the simulated tracer pathways and freshwater distribution.

4. Mechanistic link between SSH changes and gateway transport
The manuscript attributes the projected changes in export partitioning primarily to SSH changes in the eastern LIA. While correlations and regression analyses are shown, the dynamical mechanism linking SSH gradients to gateway transports is not fully demonstrated.
It would strengthen the manuscript if the authors could further clarify or illustrate the physical mechanism connecting LIA SSH changes with changes in transport through Fram Strait and the CAA. For example, diagnostics showing the associated geostrophic velocity anomalies, pressure gradients, or circulation changes along the relevant pathways would help demonstrate this mechanism more explicitly.

5. Surface geostrophic flow
The manuscript frequently refers to changes in "surface geostrophic flow" associated with SSH anomalies in the eastern LIA. However, it is not entirely clear whether geostrophic velocities were explicitly diagnosed from SSH gradients or whether these statements refer to the modeled velocity field shown in Figure 1. Clarifying this point in the text would help the reader understand how the geostrophic interpretation is derived.

6. Link between Beaufort Gyre freshwater variability and eastern LIA sea level
The manuscript suggests that freshwater accumulation and subsequent release from the Beaufort Gyre contributes to increasing freshwater content and SSH in the eastern LIA. However, the diagnostics presented mainly show spatial patterns and regional averages of freshwater content and SSH.
It would be helpful if the authors could clarify the physical pathway linking Beaufort Gyre freshwater anomalies to SSH changes north of Greenland. For example, diagnostics illustrating changes in freshwater transport or circulation pathways (e.g., along the Transpolar Drift) could help support this interpretation.

7. Interpretation of salinity increase north of Greenland
The manuscript states that more saline Eurasian waters penetrate north of Greenland, eventually contributing to the decrease in SSH in the eastern LIA. However, the supporting figure referenced (Fig. S3) shows a time-depth plot of salinity averaged over the LIA region and does not clearly indicate the origin of these waters.
The authors may wish to clarify how the intrusion of Eurasian waters is diagnosed and whether additional diagnostics (e.g., tracer analysis or circulation maps) support this interpretation.

8. Clarification of LIA vs eastern LIA terminology
The manuscript defines both the Last Ice Area (LIA) and the eastern LIA in the figures and accompanying description. However, several of the key diagnostics—particularly the comparisons involving sea surface height (SSH) and Arctic export partitioning—appear to use SSH calculated specifically over the eastern LIA (north of Greenland).
After these comparisons are introduced, the text frequently refers simply to "LIA", without specifying whether the metric being discussed corresponds to the entire LIA domain or only the eastern LIA region used in the analysis. This creates some ambiguity for the reader when interpreting the results.
Because the central argument of the manuscript relies on SSH variations in this region, I recommend that the authors consistently indicate when results correspond specifically to the eastern LIA, rather than the broader LIA domain, particularly in sections discussing correlations with export partitioning.

9. Definition of analysis periods and terminology
Several time periods are discussed in the manuscript (e.g., historical, warming scenario, late-century conditions), but these are not explicitly defined when first introduced. As a result, the reader is often required to infer the periods from the figures. For example, 1981–2000, 2051–2070, and 2081–2100 appear to represent the historical, mid-century, and late-century periods respectively. Terms such as "warming scenario," "climate warming," and "warming continues" are used throughout the text but are not explicitly linked to these time intervals.
I recommend clearly defining the analysis periods before discussing the figures and using consistent terminology throughout the manuscript.

Minor Comments

Sentence structure
Some sentences in the manuscript are overly long and could benefit from restructuring for clarity. Breaking these into shorter sentences would improve readability.
Examples include:
- Lines 51–58
- Lines 74–81

Figure referencing
Some figure references are potentially confusing or inconsistent.
For example:
- Line 174 references Fig. 1a–i, although the statement appears to refer only to specific variables. The reference should be adjusted to reflect the panels actually discussed.

- Ensure that figure references clearly correspond to the variables being described.

Specific wording corrections
Line 100: Replace “CAA transport” with “transport through the CAA”.
Line 115: Replace “an” with “in”.
Lines 528–533: it is not entirely clear whether the discussion refers to model output or observational data. Clarifying this distinction would improve readability.
Line 536: Replace “contributes” with appropriate grammatical correction if needed in context.

Figures
Figure 2a: If the figure refers specifically to the eastern LIA, the y-axis label should reflect this.
Figure 2c: Clarify the meaning of positive and negative transport values with respect to flow direction.
Figure 3d: The acronyms corresponding to the different models shown in the legend should be defined in the figure caption.
Figure 6: Please indicate the locations of the release gateways used for the dye tracers.
Fig. S3: In the comparison between the reference simulation and the wind-replaced simulation (Fig. S3), the difference between the two panels is difficult to visually interpret. A panel showing the difference between the two simulations could help illustrate the impact of wind forcing more clearly.

Methods
Line 760: Clarify the source of the river runoff forcing used in the simulations. Additionally, please discuss whether the absence of Greenland meltwater forcing may affect the results.
Line 767: The statement that the model reproduces Arctic hydrography and freshwater content “reasonably well” should specify compared to which dataset (e.g., PHC3 observations).
Lines 810–811: Please clearly define the region referred to as the “Arctic” in the wind-replacement experiment.
Lines 875–885: Provide references or documentation for the simulations used.

Reviewer #2

(Remarks to the Author)
I co-reviewed this manuscript with one of the reviewers who provided the listed reports. This is part of the Nature Communications initiative to facilitate training in peer review and to provide appropriate recognition for Early Career Researchers who co-review manuscripts.

Reviewer #3

(Remarks to the Author)
Review of “Sea level rise and fall in Last Ice Area reorganize Arctic export to the North Atlantic” by Wang et al

Summary

The authors are using a global model with a high horizontal resolution in the North Atlantic and the Arctic (4.5 km), and a courser resolution (1 degree) elsewhere, forced by atmospheric forcing from the FIO-ESM Earth System Model, to investigate how the freshwater export changes with climate change, and relates it to changes in SSH. They find in their high-resolution model that the partitioning of export between the Fram strait and the Canadian Arctic Archipelago (ACC) can be related to the SSH in the LIA north of Greenland, and nicely demonstrate the partitioning with tracer experiments. Interestingly, although the lower resolution CMIP6 models usually exhibit large biases in the Arctic, they find a similar relationship in these, corroborating the results of their high-resolution model.

Major comments:
My expertise is mainly in marine biogeochemical modelling and the links between marine biogeochemistry and ocean dynamics. Although I find that the authors have presented a well composed study with a clear story line, combining high resolution and CMIP models with observations, I cannot judge on the novelty of the main story of this manuscript.
My main comment is on the links the authors mention several times to ocean biogeochemistry. I find that the way authors are mentioning links to biogeochemistry in the abstract, makes the reader think that they will be looking deeper into this in the MS. (This was the case when I accepted to review the paper) . One way to avoid this is to remove it from the nutrients and carbon from the first sentence, and/or that in the last sentence you write “which could impact North Atlantic salinity patterns and biogeochemistry”.

The other option would be to embed this into the paper. I have not read any papers myself on how different the chemical composition is of the waters exiting the Fram Strait and the Davis strait – I would guess that there is little data. I’m wondering if the authors have found some data on the concentration of nutrients and DIC in these waters? Could you consider estimating impacts of changes in the partitioning on the export of biogeochemical tracers? CMIP models also have nutrient and DIC concentrations, - considering that they are capturing the effect of changes in SSH in the LIA on the partitioning, could you make an estimate of the change in export of carbon and nutrients? I would guess that you do not have a biogeochemical model coupled to your high-resolution model due to computational constraints?

Minor Comments

L49-51: this first sentence sounds a bit strange: Climate and ecosystems are very general (sounds like global), while then you talk about exports to the North Atlantic, also not specifying what exports you are talking about make the reader wonder about this. Consider rewriting

L59: do you mean dissolved organic carbon?

L61-65: “Once exported into the North Atlantic,
Arctic water enriched in freshwater, nutrients, and carbon can alter biogeochemical
processes in downstream regions [10–12]” : to what extent are these waters actually enriched in carbon? DIC tend to be lower in waters with lower salinity. Are you referring to dissolved organic carbon (see for example https://agupubs.onlinelibrary.wiley.com/doi/full/10.1029/2020GL089686)

L84-88: It is unclear from the writing whether the references have mentioned this hypothesis, or if the hypothesis is something you have come up with based on the facts in these two references (as it is written it sounds like the partitioning of tracers between the two gateways and their impact has been discussed in the two references)

L318-319: Consider removing the sentence on the transport through the Bering strait and Barents Sea Opening, it somewhat distracts the reader from the focus of the story

L356: variability components -> natural variability components?

Figure 2c: consider moving to supplementary material? Same information is to some extent in a and b.

Fig 2c: What is the correlation coefficient of the to time-series? Shouldn't they be completely anti-correlated?

Fig3 : I would consider only keeping a) and b) in the main MS, and moving the other to the supplementary. In a) I would suggest remove the line showing the temperature, for clarity.

L810-812: To reduce computational cost and improve efficiency, high resolution was applied only in the Arctic. Earlier you wrote that the high resolution was applied also in the North Atlantic. If you do not have high resolution in the North Atlantic, how would that affect your results when it comes to the impact of the SSH in the North Atlantic Subpolar Gyre on the flow partitioning?

L875-884: please add references for the ESMs.

L930: did you use different reference salinities for the different CMIP models, which have different mean salinities of the Arctic Ocean?

Reviewer #4

(Remarks to the Author)
Review of manuscript

Sea level rise and fall in Last Ice Area reorganize
Arctic export to the North Atlantic
by Qiang Wang, and co-authors

The authors address an important and timely aspect of changes in Arctic sea ice and freshwater content and export in the context of anthropogenic climate change. There has been considerable interest in this subject, and there have been a number of papers published, based on modelling and/or observations. These papers are well referenced in the present manuscript.
This new study addresses a potentially important aspect that has been largely missing from previous studies: namely the effect of dynamic sea-level variability. As such, it can add to the literature and be a novel contribution that advances significantly enough to be a suitable publication in a geophysical sciences journal.
However, after having read this manuscript three times, this reviewer is not convinced this to be a Nature Communications publication in its present form. I have both reviewed and co-authored several papers submitted to Nature Comms. and comparable journals such as Science Advances. I believe that this could be an excellent contribution to a more specialized journal, such as Climate Dynamics, Journal of Geophysical Research (Oceans) or perhaps one of the Nature partner journals.
I believe that it needs at least a “major revisions” by re-writing to improve the significance of the results and adjust to this journal’s broad readership. Therefore, I recommend that in its present form, this submission should be returned to the authors. However, I would encourage the authors to consider to re-think and re-write before submitting a new version to Nature Comms. or one of the many excellent, more specialized journals such as those noted above.

Comments on the content and presentation:
The authors have done a very extensive analysis, and this reviewer has confidence in the soundness of the results, especially for the recent and near future scenarios. There are however so many uncertainties in what the latter part of the

21st century with bring, because of different global warming rates due differences in climate sensitivity and different emissions scenarios – as well as how a future AMOC weakening or “collapse” would affect the sea ice and freshwater in the Arctic.
In my opinion, the order of the presentation could be adjusted, as follows After briefly introducing the hypothesis and the suggestions from the modelling projections, which will be presented in detail afterwards, first present the observational variability and trends (and the ability of the model to simulate this, i.e., sometime after lines 105-112, and show Figure 5c,d as the new Figure 1a,b. Then follow this with the panels in Fig5a,b and e,f – i.e., show these six panels in the new Figure 1,
The figures are informative, both spatially and temporally, and generally well done, with further results relegated to the Supplementary materials. Figure 2 is of central importance, and the consequences of circulation changes are well illustrated spatially. For example, Figure 6a and especially 6b are amongst the most interesting results that of most importance for the downstream impacts in the North Atlantic, (although these impacts are not well developed in the paper). I think that however some of the multi-panel figures are too “busy”, particularly Figure 5.
Regarding the section “Changing LIA dynamic sea level”: Is the sea ice in the LIA important in this context? That is, does the evolution of the sea ice in this region matter, or it really the broader Beaufort Gyre and Arctic Ocean that controls the circulation in the region?
Regarding the conclusion: “The recently emerging sea level rise in the LIA indicates that the Arctic-Atlantic linkage could already be tracking the projected trajectory toward a regime dominated by Fram Strait export.” The freshwater export (solid and liquid – same ingredients, different mix) has been long established to be dominated by Fram Strait, so what is the significance of these new results? If it is the liquid freshwater transported through Fram Strait that is becoming dominant versus the CAA, then this should be stated clearly.

Presentation quality:
The manuscript is thoroughly referenced, and writing quality is adequate throughout. In addition to telling the story better for a broader readership, there are several minor writing improvements needed for further clarity. For example:
Title: It should say “…Arctic Ocean freshwater export...”
Abstract (and elsewhere): It should be made immediately clear that these are modelling outcomes. “Here we show that….” can be rephrased as “Here we present modelling results that show that….”

Version 1:

Reviewer comments:

Reviewer #1

(Remarks to the Author)
General Assessment

The authors addressed the major concerns raised in the initial review through substantial revisions to both the analysis and presentation of the manuscript. In particular, they strengthened the mechanistic interpretation linking dynamic sea level changes in the eastern Last Ice Area to Arctic export redistribution by adding new circulation diagnostics, clarified methodological aspects related to model resolution and gateway transports, and improved the organization and terminology throughout the paper. Additional validation, sensitivity analyses, and new supplementary figures were also included to better support the robustness of the results and their broader climatic implications. Overall, the revised manuscript is significantly improved in clarity, rigor, and presentation.

The remaining vulnerabilities are primarily related to the strength of the causal interpretation relative to the analyses presented. The manuscript sometimes uses very strong wording such as “governs,” “determines,” or “dominates,” even though the conclusions are mainly supported by correlations, regression analyses, and sensitivity experiments rather than a full dynamical attribution framework. While the proposed mechanism is scientifically convincing and well supported, some statements may imply a stronger level of causal proof than has been formally demonstrated. To strengthen the manuscript before publication, the authors could slightly soften some of the causal language (e.g., replacing “governs” with “strongly influences” or “is a primary control on”), which would make the interpretation more balanced and more robust to reviewer or editorial scrutiny without weakening the scientific impact of the study.

Minor comments
Line 82: first mention of “eastern LIA” without proper geographic reference. Add “(Fig. 2a)”.
Line 95: define FWC (freshwater content) here. No need to do it multiple times throughout the manuscript. The authors alternate quickly between full term and acronym in adjacent sentences. Not wrong, but slightly repetitive in some sections.
Line 135: be consistent with hyphenation, use “mid-century” or “mid century”, not both.
Line 168–169: wording may be slightly too strong: “This evolution determines…” and “major control on…” could be softened slightly to better match the evidence presented.
Line 190: “The regression coefficient… is about 70% larger…” This is okay mathematically, but stylistically could be:

“approximately 70% larger”
Line 191: “exerts the dominant influence” may sound stronger than the regression analysis alone can fully demonstrate.
Line 202: “David Strait”, should be “Davis Straid”
Line 298–300: “key region governing…” and “largely determines…” may again benefit from slightly softer causal wording
Line 307-308: sentence reads awkwardly. A suggestion could be “The Arctic supplies freshwater to the subpolar North Atlantic (partly through sea ice export) primarily via Fram Strait.”
Line 342: “…, about 30 km wide at its narrowest section”
Line 433–434: “supporting a dominant dynamical role…” could potentially be softened to “supporting an important dynamical role…”.
Fig. 6 caption: “Solid strait lines in (b-d) denote linear fits.” Should be: “Solid straight lines…”
Fig. 2 caption: “The plots highlight recent changes…” Suggestion: “The panels highlight…”

Reviewer #2

(Remarks to the Author)
I co-reviewed this manuscript with one of the reviewers who provided the listed reports. This is part of the Nature Communications initiative to facilitate training in peer review and to provide appropriate recognition for Early Career Researchers who co-review manuscripts.

Reviewer #3

(Remarks to the Author)
I thank the authors for the work they have done on the MS. They have answered all my comments and I only have a one minor remaining:

I think that the examples that you bring up in “(e.g., enhanced acidification and mixing with riverine dissolved organic carbon)”, are quite specific, and may not be very representative. Could you just write "due to biogeochemical processes and mixing with other watermasses?"

Open Access This Peer Review File is licensed under a Creative Commons Attribution 4.0 International License, which permits use, sharing, adaptation, distribution and reproduction in any medium or format, as long as you give appropriate credit to the original author(s) and the source, provide a link to the Creative Commons license, and indicate if changes were made.
In cases where reviewers are anonymous, credit should be given to 'Anonymous Referee' and the source.
The images or other third party material in this Peer Review File are included in the article’s Creative Commons license, unless indicated otherwise in a credit line to the material. If material is not included in the article’s Creative Commons license and your intended use is not permitted by statutory regulation or exceeds the permitted use, you will need to obtain permission directly from the copyright holder.
To view a copy of this license, visit https://creativecommons.org/licenses/by/4.0/

Reviewer #1

Dear reviewer,

Thank you very much for reviewing our paper and providing very helpful comments. We revised the paper following your comments. Please see our responses in detail below.

General Assessment

This manuscript investigates how dynamic sea level changes in the Last Ice Area (LIA) influence the partitioning of Arctic freshwater export between Fram Strait and the Canadian Arctic Archipelago (CAA) using high-resolution ocean–sea ice simulations. The study addresses an important problem for Arctic–North Atlantic coupling and presents an interesting mechanism linking LIA sea level variability with export redistribution.

Overall, the manuscript is clearly written, and the topic is relevant to the Arctic climate community. However, several aspects require clarification or strengthening and I recommend that it be returned for a major revision. In particular:

• The definition of analysis periods and terminology used throughout the manuscript needs clarification.
• The manuscript relies heavily on supplementary figures, including key model evaluation results.
• Several figures require clearer explanations, especially regarding transport sign conventions.
• Some methodological aspects of the model configuration and forcing need further clarification.
• The dynamical mechanism linking sea level changes to gateway transports could be more clearly demonstrated.
• Line numbers appear misaligned throughout the manuscript, which makes it difficult to reference specific parts of the text during review. Please correct this formatting issue.

Below I outline several major and minor comments intended to help improve the clarity and robustness of the manuscript.

Thank you for your recognition of our work and for your comments, which helped to improve the manuscript. We revised the paper following your suggestions. See our reply to the individual comments below.
For the issue of line numbering, we were using the default line numbering of the provided Latex template. Now we use customer line numbering, which solves the issue except for the abstract part.

Major Comments

1. Manuscript relies heavily on supplementary figures

Several key arguments in the manuscript rely on figures located in the Appendix or Supplementary material. In particular, the evaluation of the model's representation of Arctic freshwater distribution and hydrography is presented primarily in Fig. S1.

This comparison is essential because it forms the basis for the authors' claim that the high-resolution simulation realistically reproduces Arctic hydrography and therefore can "provide future projections with reduced uncertainty." However, this evaluation is only briefly described in the text and relies on a figure not included in the main manuscript. I recommend including at least a condensed version of this model validation in the main text (or moving the relevant figure from the Supplementary material), as it is critical for establishing the credibility of the simulations.

We move Fig. S1 to the main text (as Fig. 3 now) following your suggestion.

However, we would like to note that reviewer 3 suggested moving some of the main figure panels to supplementary and reviewer 4 suggested revising the paper to make it read more like a NC paper. Indeed, it is common that figures related to validation and supporting roles are placed in supplementary material for this journal, keeping only key results in the main text. We would like to make all reviewers happy, but we would need to negotiate and compromise in the case of somehow contradicting suggestions. The final paper structure will thus depend on the agreement among all reviewers and the editor. We hope that you can understand the situation.

2. Representation of transport through the Canadian Arctic Archipelago

The partitioning of Arctic export depends strongly on the representation of flow through the Canadian Arctic Archipelago. The model uses a horizontal resolution of approximately 4.5 km in the Arctic, which may still be marginal for resolving the narrowest CAA channels.

The authors should clarify:

- How well the model grid resolves the major CAA passages.
- Whether transport through the narrowest channels is fully resolved or parameterized.
- Whether any validation of simulated CAA transport against observations has been performed.

Providing additional information on this point would help assess the robustness of the export partitioning results.

We add a comparison with observational estimates of volume and freshwater transports (Supplementary Fig. 1). The model has a decent representation for transports through CAA and other Arctic gateways.

"Furthermore, the simulation reasonably reproduces ocean volume and freshwater transports through Arctic gateways estimated from observations (Supplementary Fig. 1). The 4.5 km horizontal resolution can adequately resolve the two major CAA channels

(Parry Channel, about 50 km wide at its narrowest point; Nares Strait, about 30 km wide), but not the smaller straits (Hell Gate and Cardigan Strait, with a total width of about 12 km) [25]. Overall, the simulated total transports through the CAA as measured at Davis Strait are consistent with the observational estimates and largely fall within the uncertainty range (Supplementary Fig. 1)." (Lines 338-346)

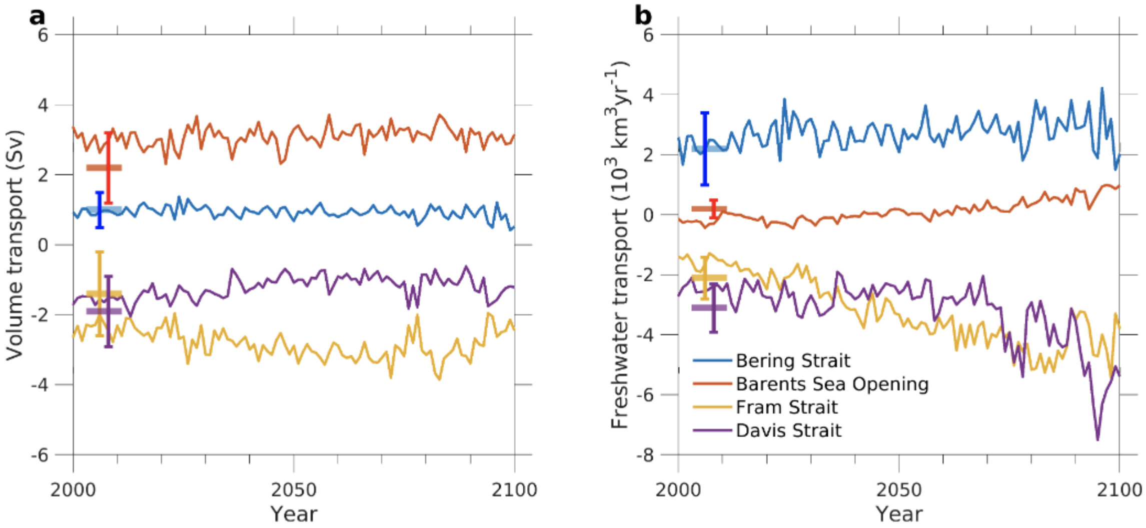

Supplementary Fig. 1: Volume and freshwater transports through Arctic gateways in the high-resolution simulations. (**a**) Volume transports. (**b**) Freshwater transports. The synthesized values obtained by combining inverse modeling and observations (Tsubouchi et al., 2024) are shown as thick lines with ranges of standard deviations. Negative values indicate export from the Arctic.

3. Resolution asymmetry (Arctic vs North Atlantic)

The model employs high resolution in the Arctic (~4.5 km), but the resolution in the North Atlantic is coarser (~24 km), which does not resolve mesoscale eddies. Since the redistribution of Arctic-sourced waters in the subpolar gyre is influenced by eddy-driven transport, the authors may wish to discuss more explicitly how this limitation could affect the simulated tracer pathways and freshwater distribution.

In the simulations, eddy parameterizations are activated where mesoscale eddies are not resolved, so the first order effects of eddies are already represented in the simulations. We clarified this point in the revised manuscript to avoid misleading:

"We note that, to reduce computational cost and enable multiple century-long simulations, a 4.5 km resolution was applied only in the pan-Arctic region, including the Canadian Arctic Archipelago. The resolution in the North Atlantic is insufficient to explicitly resolve mesoscale eddies; therefore, eddy parameterizations were applied to represent their effects on tracer transport and mixing. The SSH variability in the

subpolar gyre is well reproduced in comparison with satellite observations (Fig. 1c)." (Lines 390-395)

4. Mechanistic link between SSH changes and gateway transport

The manuscript attributes the projected changes in export partitioning primarily to SSH changes in the eastern LIA. While correlations and regression analyses are shown, the dynamical mechanism linking SSH gradients to gateway transports is not fully demonstrated.

It would strengthen the manuscript if the authors could further clarify or illustrate the physical mechanism connecting LIA SSH changes with changes in transport through Fram Strait and the CAA. For example, diagnostics showing the associated geostrophic velocity anomalies, pressure gradients, or circulation changes along the relevant pathways would help demonstrate this mechanism more explicitly.

We add a figure to show that the upper ocean circulation mainly follows surface geostrophic currents.

"Surface geostrophic currents derived from SSH closely represent upper-ocean circulation patterns (cf. Fig. 4a-c and Supplementary Fig. 2), while changes in SSH are mainly determined by changes in FWC (Fig. 4d-f and Fig. 4g-i)." (Lines 136-139)

As we presented in the paper, the flow streamlines of both the upper ocean mean velocity (Fig. 4a-c) and the surface geostrophic currents (Supplementary Fig. 2a-c) illustrate the changes in the orientation of the flow at the end of the Transpolar Drift.

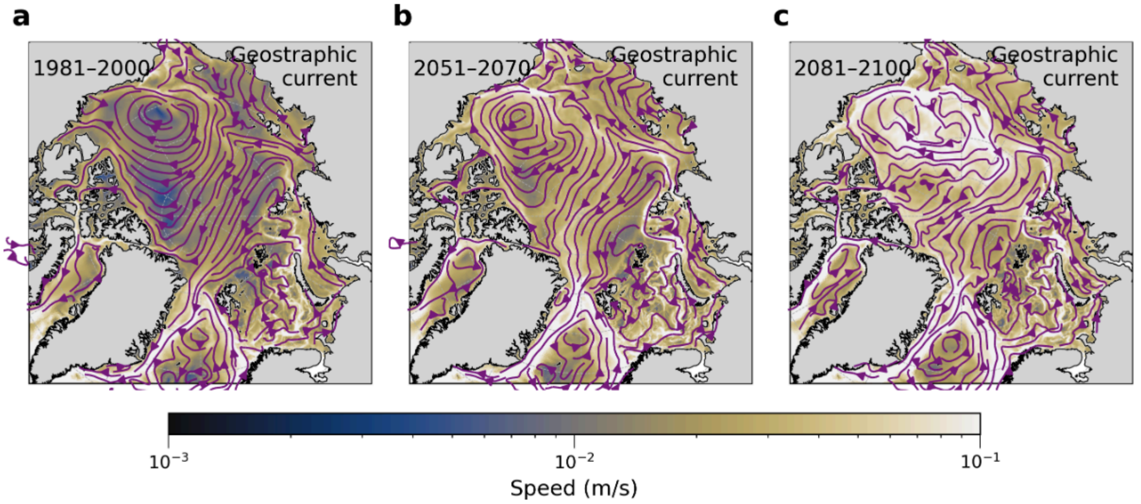

Supplementary Fig. 2: Surface geostrophic currents. (**a-c**) Surface geostrophic currents derived from sea surface height in different periods: (a) 1981–2000, (b) 2051–2070, (c) 2081-2100. Colors show speeds and arrows indicate current directions. The results indicate that changes in the upper ocean circulation shown in Fig. 4a-c are mainly determined by changes in surface geostrophic currents.

Editorial Note: Fig R1 in this Peer Review File is reproduced under a CC BY 4.0 license: https://creativecommons.org/licenses/by/4.0/

5. Surface geostrophic flow

The manuscript frequently refers to changes in “surface geostrophic flow” associated with SSH anomalies in the eastern LIA. However, it is not entirely clear whether geostrophic velocities were explicitly diagnosed from SSH gradients or whether these statements refer to the modeled velocity field shown in Figure 1. Clarifying this point in the text would help the reader understand how the geostrophic interpretation is derived.

See our reply to the last comment. We were showing the ocean velocity averaged over the upper ocean in original Fig. 1, but the circulation is largely determined by surface geostrophic currents from SSH gradients.

6. Link between Beaufort Gyre freshwater variability and eastern LIA sea level

The manuscript suggests that freshwater accumulation and subsequent release from the Beaufort Gyre contributes to increasing freshwater content and SSH in the eastern LIA. However, the diagnostics presented mainly show spatial patterns and regional averages of freshwater content and SSH.

It would be helpful if the authors could clarify the physical pathway linking Beaufort Gyre freshwater anomalies to SSH changes north of Greenland. For example, diagnostics illustrating changes in freshwater transport or circulation pathways (e.g., along the Transpolar Drift) could help support this interpretation.

The relationship between Beaufort Gyre freshwater and that north of Greenland has been studied in one of our old papers by adding dye tracers into the Beaufort Gyre (shown in the adopted figure below). We cited the work in the paper:

“Since then, part of this excess freshwater has been released under a cyclonic wind anomaly over the Canada Basin, increasing freshwater content in the eastern LIA (Fig. 1b) and thereby elevating SSH in that region (Fig. 1d and Fig. 2) [36]. ” (Lines 94-96)

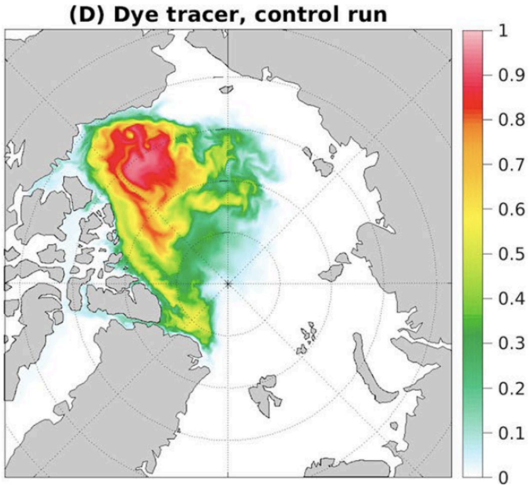

Fig R1: Concentration of dye tracer four years after being injected into the upper Beaufort Gyre. It indicates that released freshwater fills the LIA before export. Adopted from Ref 36.

[36] Wang, Q., Danilov, S. & Jung, T. Arctic freshwater anomaly transiting to the North Atlantic delayed within a buffer zone. Nat. Geosci. 17, 1218–1221 (2024). https://doi.org/10.1038/s41561-024-01592-1

7. Interpretation of salinity increase north of Greenland

The manuscript states that more saline Eurasian waters penetrate north of Greenland, eventually contributing to the decrease in SSH in the eastern LIA. However, the supporting figure referenced (Fig. S3) shows a time-depth plot of salinity averaged over the LIA region and does not clearly indicate the origin of these waters.

The authors may wish to clarify how the intrusion of Eurasian waters is diagnosed and whether additional diagnostics (e.g., tracer analysis or circulation maps) support this interpretation.

We added a figure showing ocean circulation at 100 m depth, where the reversal of the salinity trend is clearly evident in the time-depth salinity plot. The figure indicates that during the first two periods, Eurasian waters at this depth are primarily advected toward Fram Strait, whereas in 2081–2100 it is redirected toward the region north of Greenland.

"By the late 21st century, … More saline waters from the Eurasian Basin are advected toward the region north of Greenland (Fig. 4c and Supplementary Fig. 3), increasing salinity (Supplementary Fig. 4a) and thus lowering SSH in the eastern LIA (Fig. 4f)." (Lines 162-165)

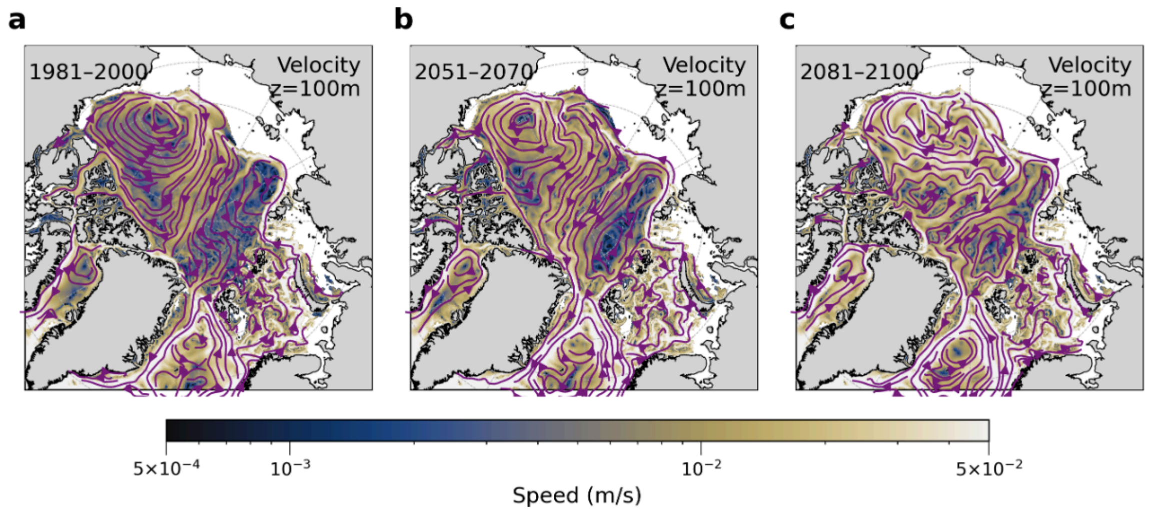

Supplementary Fig. 3: Ocean currents at 100 m depth. (**a-c**) Ocean currents at 100 m depth in different periods: (a) 1981–2000, (b) 2051–2070, (c) 2081-2100. Colors show speeds and arrows indicate current directions. Eurasian waters are advected toward the eastern Last Ice Area north of Greenland in the period of 2081–2100.

8. Clarification of LIA vs eastern LIA terminology

The manuscript defines both the Last Ice Area (LIA) and the eastern LIA in the figures and accompanying description. However, several of the key diagnostics—particularly the comparisons involving sea surface height (SSH) and Arctic export partitioning—appear to use SSH calculated specifically over the eastern LIA (north of Greenland).

After these comparisons are introduced, the text frequently refers simply to “LIA”, without specifying whether the metric being discussed corresponds to the entire LIA domain or only the eastern LIA region used in the analysis. This creates some ambiguity for the reader when interpreting the results.

Because the central argument of the manuscript relies on SSH variations in this region, I recommend that the authors consistently indicate when results correspond specifically to the eastern LIA, rather than the broader LIA domain, particularly in sections discussing correlations with export partitioning.

We removed the figure and text about comparing the eastern LIA and the whole LIA during the revision, which are not important and may lead to confusion. We have only one definition now, the “eastern LIA”.

9. Definition of analysis periods and terminology

Several time periods are discussed in the manuscript (e.g., historical, warming scenario, late-century conditions), but these are not explicitly defined when first introduced. As a result, the reader is often required to infer the periods from the figures.

For example, 1981–2000, 2051–2070, and 2081–2100 appear to represent the historical, mid-century, and late-century periods respectively. Terms such as “warming scenario,” “climate warming,” and “warming continues” are used throughout the text but are not explicitly linked to these time intervals.
I recommend clearly defining the analysis periods before discussing the figures and using consistent terminology throughout the manuscript.
We add the definition of the analyzed periods: (Lines 132-136)
“Projected changes in Arctic freshwater content, SSH and upper-ocean circulation in the high-resolution simulation are shown in Fig. 4. Three periods are considered to characterize these changes: the historical period (1981–2000), the mid-century period (2051–2070), and the late-century period (2081–2100).” and try to use the same terminology in the paper when possible.

Minor Comments

Sentence structure

Some sentences in the manuscript are overly long and could benefit from restructuring for clarity. Breaking these into shorter sentences would improve readability.
Examples include:
- Lines 51–58 (L34-38)
- Lines 74–81 (L48-52)

We shortened these overly long sentences to improve readability.

Figure referencing
Some figure references are potentially confusing or inconsistent.
For example:
- Line 174 references Fig. 1a–i, although the statement appears to refer only to specific variables. The reference should be adjusted to reflect the panels actually discussed.
- Ensure that figure references clearly correspond to the variables being described.

We checked the references to figure panels to make them consistent.

Specific wording corrections

Line 100: Replace “CAA transport” with “transport through the CAA”.
Changed. (L63)
Line 115: Replace “an” with “in”.
It should be “an”.
Lines 528–533: it is not entirely clear whether the discussion refers to model output or observational data. Clarifying this distinction would improve readability.

We added “These changes are consistently captured by available observations and the hindcast simulation (Figs. 1a-d).” to make it clear. (L96-97)

Line 536: Replace “contributes” with appropriate grammatical correction if needed in context.

Changed to “contributed” (L100)

Figures

Figure 2a: If the figure refers specifically to the eastern LIA, the y-axis label should reflect this.

Revised. (Fig. 5a)

Figure 2c: Clarify the meaning of positive and negative transport values with respect to flow direction.

We added “Negative values indicate Arctic outflow.” in the figure caption. (Fig. 5)

Figure 3d: The acronyms corresponding to the different models shown in the legend should be defined in the figure caption.

They are the names of the models registered for CMIP6.

Figure 6: Please indicate the locations of the release gateways used for the dye tracers.

Locations added in the figure. (Fig. 8)

Fig. S3: In the comparison between the reference simulation and the wind-replaced simulation (Fig. S3), the difference between the two panels is difficult to visually interpret. A panel showing the difference between the two simulations could help illustrate the impact of wind forcing more clearly.

A panel showing the difference is added. (Supplementary Fig. 4c)

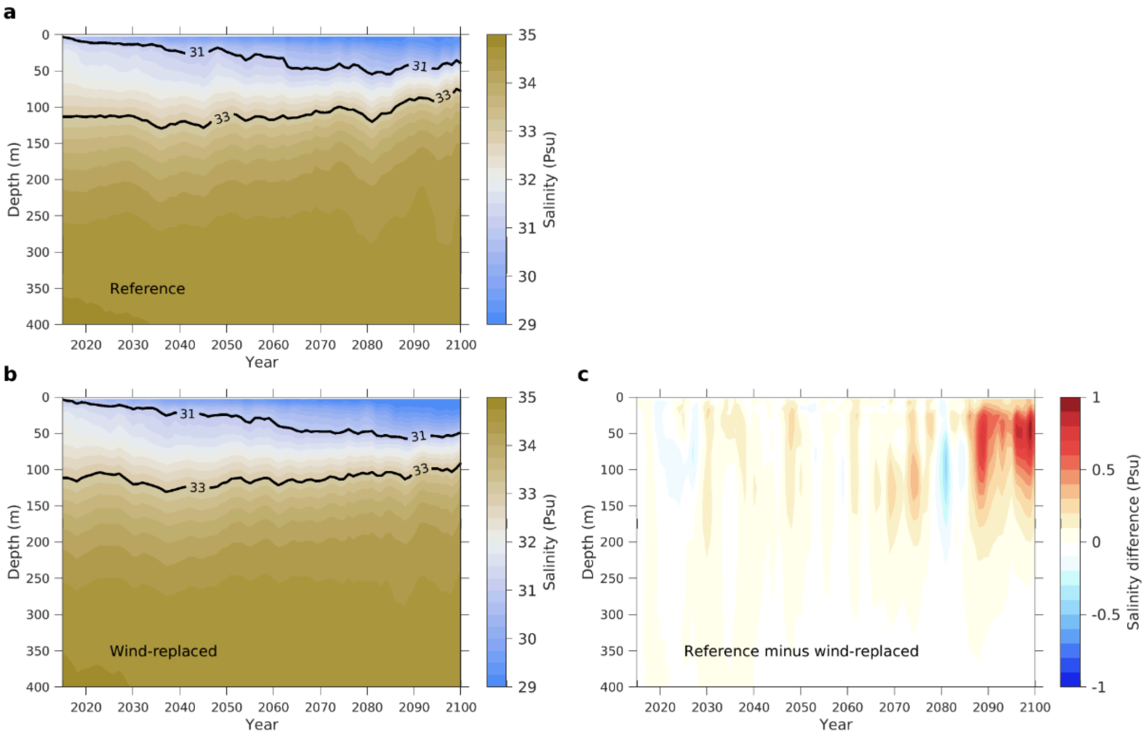

Supplementary Fig. 4: Evolution of salinity averaged over the eastern Last Ice Area (LIA). (**a**) Time-depth plot of salinity in the reference future projection simulations using high-resolution FESOM2. (**b**) Same as (a), but for the wind-replaced simulations, in which Arctic winds are replaced by 20th-century winds. (**c**) Difference between the two simulations.

Methods

Line 760: Clarify the source of the river runoff forcing used in the simulations. Additionally, please discuss whether the absence of Greenland meltwater forcing may affect the results.

We added discussions as you suggested:

"The forcing fields derived from FIO-ESM v2.1 include near-surface winds, air temperature and specific humidity, downward longwave and shortwave radiation, precipitation, and river runoff." (L328-330)

"Additionally, freshwater input from increased melting of the Greenland Ice Sheet was not included in our high-resolution simulations, consistent with CMIP6 climate model simulations. We find that the freshening and expansion of the Beaufort Gyre can drive an increase in dynamic sea level in the eastern LIA. While additional freshwater input may modify the magnitude of these changes and influence ocean circulation, it is unlikely to weaken Beaufort Gyre spinup or diminish its role identified here. Consequently, our main findings regarding the rise in dynamic sea level and the redirection of Arctic export toward Fram Strait are likely to remain qualitatively robust.

Nevertheless, quantifying the effects of land ice meltwater requires dedicated investigation in future studies.” (403-412)

Line 767: The statement that the model reproduces Arctic hydrography and freshwater content “reasonably well” should specify compared to which dataset (e.g., PHC3 observations).
Reference is added. (L335)

Lines 810–811: Please clearly define the region referred to as the “Arctic” in the wind-replacement experiment.
Definition added. “... (defined by the Bering Strait, Barents Sea Opening, Fram Strait, and the northern boundary of the Canadian Arctic Archipelago) …” (L367-369)

Lines 875–885: Provide references or documentation for the simulations used.
A reference list is added in Supplementary Table 1.

Reviewer #2

Dear reviewer,

Thank you very much for helping review our paper and provide very helpful comments. We hope that the revised paper addressed your comments.

I co-reviewed this manuscript with one of the reviewers who provided the listed reports. This is part of the Nature Communications initiative to facilitate training in peer review and to provide appropriate recognition for Early Career Researchers who co-review manuscripts.

Reviewer #3

Dear reviewer,

Thank you very much for reviewing our paper and providing very helpful comments. We revised the paper following your comments. Please see our responses in detail below.

Review of "Sea level rise and fall in Last Ice Area reorganize Arctic export to the North Atlantic" by Wang et al

Summary

The authors are using a global model with a high horizontal resolution in the North Atlantic and the Arctic (4.5 km), and a courser resolution (1 degree) elsewhere, forced by atmospheric forcing from the FIO-ESM Earth System Model, to investigate how the freshwater export changes with climate change, and relates it to changes in SSH. They find in their high-resolution model that the partitioning of export between the Fram strait and the Canadian Arctic Archipelago (ACC) can be related to the SSH in the LIA north of Greenland, and nicely demonstrate the partitioning with tracer experiments. Interestingly, although the lower resolution CMIP6 models usually exhibit large biases in the Arctic, they find a similar relationship in these, corroborating the results of their high-resolution model.

Major comments:
My expertise is mainly in marine biogeochemical modelling and the links between marine biogeochemistry and ocean dynamics. Although I find that the authors have presented a well composed study with a clear story line, combining high resolution and CMIP models with observations, I cannot judge on the novelty of the main story of this manuscript.
My main comment is on the links the authors mention several times to ocean biogeochemistry. I find that the way authors are mentioning links to biogeochemistry in the abstract, makes the reader think that they will be looking deeper into this in the MS. (This was the case when I accepted to review the paper) . One way to avoid this is to remove it from the nutrients and carbon from the first sentence, and/or that in the last sentence you write "which could impact North Atlantic salinity patterns and biogeochemistry".
We revised the first sentence in the abstract to avoid confusion: "Arctic freshwater is exported to the North Atlantic through Fram Strait and the Canadian Arctic Archipelago, and its partitioning influences subpolar ocean stratification." We keep the implication wording in the last sentence. Also see our reply below.

The other option would be to embed this into the paper. I have not read any papers myself on how different the chemical composition is of the waters exiting the Fram Strait and the Davis strait – I would guess that there is little data. I'm wondering if the authors have found some data on the concentration of nutrients and DIC in these waters? Could you consider estimating impacts of changes in the partitioning on the export of biogeochemical tracers? CMIP models also have nutrient and DIC concentrations, - considering that they are capturing the effect of changes in SSH in the LIA on the partitioning, could you make an estimate of the change in export of carbon and nutrients? I would guess that you do not have a biogeochemical model coupled to your high-resolution model due to computational constraints?
It is known that current coarse-resolution Earth System Models face challenges in adequately representing biogeochemical processes (Bopp et al., 2013; Kwiatkowski et al., 2020), not only in terms of tracer fluxes through Arctic gateways but more generally. There is also no biogeochemical model component in the simulations we performed. As the present study does not aim to explicitly investigate biogeochemistry, during the revision we instead conducted simulations with dye tracers released at Bering Strait to quantify the transport pathways and distribution of Pacific Water in the North Atlantic.

"The contrasting spatial distributions of the two tracers also imply potential regional ecosystem impacts, because Arctic waters differ markedly from North Atlantic waters in nutrient content and carbonate chemistry [12, 56]. Pacific Water, for instance, is characterized by relatively low salinity and high nutrient concentrations [57, 58]. It is further modified during its transit through the Arctic (e.g., enhanced acidification and mixing with riverine dissolved organic carbon) [56, 59]. To track its fate, we released an additional dye tracer at Bering Strait. In the historical period, Pacific Water is mainly exported through the CAA, and its spreading closely resembles that of the CAA tracer (Supplementary Fig. 9a). By the mid century, however, its export is increasingly redirected toward the Fram Strait, leading to a spreading pattern that more closely resembles that of the Fram Strait tracer (Supplementary Fig. 9b). These results highlight how shifts in Arctic export pathways can alter the delivery of biogeochemically distinct water masses to the subpolar North Atlantic. The consequences for regional ecosystems warrant dedicated future study." (Lines 283-296)

"To illustrate the impacts of changes in Arctic export partitioning on biogeochemically relevant matter in the subpolar North Atlantic, we introduced a dye tracer restored to unity at Bering Strait to track the fate of Pacific Water. The tracer was released for 14 years starting in 1980 and 2050, respectively. In combination with the Fram Strait and Davis Strait tracers described above, these experiments illustrate how climate change is expected to reorganize the distribution of Pacific-origin water in the subpolar North Atlantic." (Lines 358-364)

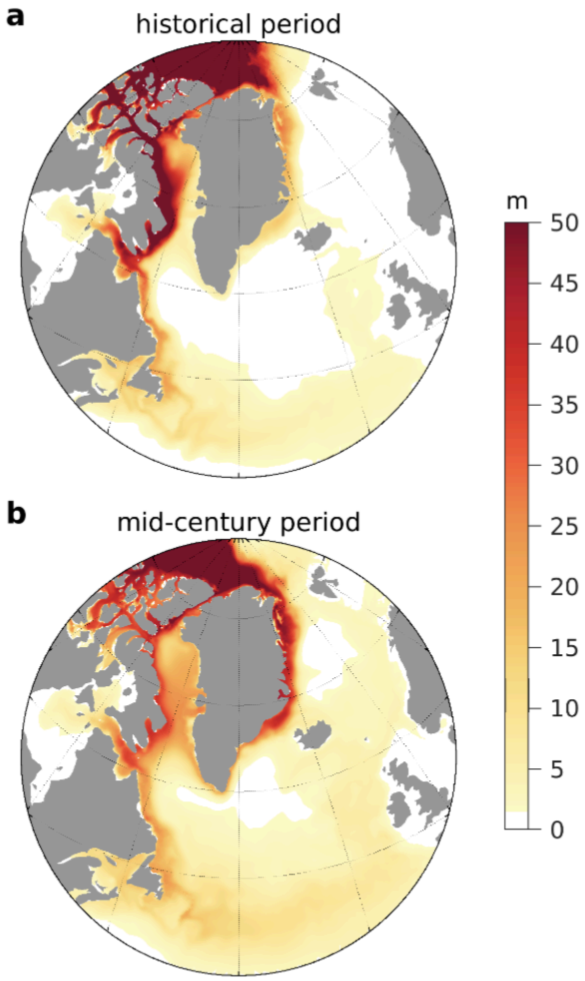

Supplementary Fig. 9: Future changes in the spatial distribution of Pacific Water. **(a,b)** Pacific Water inventory during the (a) historical and (b) mid-century periods. A dye tracer equal to one was release in the Bering Strait starting in 1980 and 2050, respectively. Shown is the vertically integrated tracer concentration, representing Pacific Water inventory, averaged over 1994 and 2064 (that is, 14 years after the start of the release). In the historical period, the spreading of Pacific Water closely resembles that of the Canadian Arctic Archipelago (CAA) tracer shown in Fig. 8b, whereas in the mid-century period it more closely resembles the Fram Strait tracer shown in Fig. 8c.

Ref:

Bopp, L., Resplandy, L., Orr, J. C., Doney, S. C., Dunne, J. P., Gehlen, M., Halloran, P., Heinze, C., Ilyina, T., Séférian, R., Tjiputra, J., and Vichi, M.: Multiple stressors of ocean ecosystems in the 21st century: projections with CMIP5 models, Biogeosciences, 10, 6225–6245, https://doi.org/10.5194/bg-10-6225-2013, 2013.

Kwiatkowski, L., Torres, O., Bopp, L., Aumont, O., Chamberlain, M., Christian, J. R., Dunne, J. P., Gehlen, M., Ilyina, T., John, J. G., Lenton, A., Li, H., Lovenduski, N. S.,

Orr, J. C., Palmieri, J., Santana-Falcón, Y., Schwinger, J., Séférian, R., Stock, C. A., Tagliabue, A., Takano, Y., Tjiputra, J., Toyama, K., Tsujino, H., Watanabe, M., Yamamoto, A., Yool, A., and Ziehn, T.: Twenty-first century ocean warming, acidification, deoxygenation, and upper-ocean nutrient and primary production decline from CMIP6 model projections, Biogeosciences, 17, 3439–3470, https://doi.org/10.5194/bg-17-3439-2020, 2020.

Minor Comments

L49-51: this first sentence sounds a bit strange: Climate and ecosystems are very general (sounds like global), while then you talk about exports to the North Atlantic, also not specifying what exports you are talking about make the reader wonder about this. Consider rewriting
We revised this sentence: "The Arctic Ocean exerts a strong influence on regional climate and marine ecosystems through its export of freshwater and chemical tracers to the North Atlantic." (L32-33)

L59: do you mean dissolved organic carbon?
Yes, the text is revised. (L39)

L61-65: "Once exported into the North Atlantic, Arctic water enriched in freshwater, nutrients, and carbon can alter biogeochemical processes in downstream regions [10–12]" : to what extent are these waters actually enriched in carbon? DIC tend to be lower in waters with lower salinity. Are you referring to dissolved organic carbon (see for examplehttps://agupubs.onlinelibrary.wiley.com/doi/full/10.1029/2020GL089686)
We changed carbon to dissolved organic carbon, and added the suggested reference. (L41-42)

L84-88: It is unclear from the writing whether the references have mentioned this hypothesis, or if the hypothesis is something you have come up with based on the facts in these two references (as it is written it sounds like the partitioning of tracers between the two gateways and their impact has been discussed in the two references)
We removed the two references as this sentence is an implication from the sentence above and not clearly discussed in those references. (L56)

L318-319: Consider removing the sentence on the transport through the Bering strait and Barents Sea Opening, it somewhat distracts the reader from the focus of the story
We removed this sentence.

L356: variability components -> natural variability components?

We added “natural”. (L197)

Figure 2c: consider moving to supplementary material? Same information is to some extent in a and b.
The anticorrelated changes at the two gateways provide an immediately accessible signal that can be readily appreciated by readers. For this reason, we would like to retain Fig. 2c (now Fig. 5c) in the main figure, if you agree.

We also note that Reviewer 1 expressed concern that the manuscript relies too heavily on supplementary figures. We have aimed to strike a balance between clarity and completeness. We hope that the current presentation with compromisation in terms of figure arrangement addresses the concerns of all reviewers.

Fig 2c: What is the correlation coefficient of the two time-series? Shouldn't they be completely anti-correlated?
They are highly anticorrelated, but not completely, because volume transports through other Arctic gateways also have interannual variability and it is the sum of the four gateways that is close to zero (not the sum of the two gateways). We add the value of correlation coefficient in the panel. (Fig. 5c)

Fig3 : I would consider only keeping a) and b) in the main MS, and moving the other to the supplementary. In a) I would suggest remove the line showing the temperature, for clarity.
We move the air temperature time series into a supplementary figure, where we compare both SSP585 and SSP245 scenarios. We prefer to keep c) and d) in the main MS to maintain a balance in the presentation, for the reason mentioned above.

L810-812: To reduce computational cost and improve efficiency, high resolution was applied only in the Arctic. Earlier you wrote that the high resolution was applied also in the North Atlantic. If you do not have high resolution in the North Atlantic, how would that affect your results when it comes to the impact of the SSH in the North Atlantic Subpolar Gyre on the flow partitioning?
The global resolution is nominal one degree, so 24 km in the northern North Atlantic is finer than average, but not eddy resolving. Eddy parameterizations were switched on where eddies are not explicitly resolved, so the first order effects of eddies are present in the model. As we show, the simulated SSH in the subpolar gyre compares well with satellite observations. We made these aspects clearer in the revised paper:
“The resolution in the North Atlantic is insufficient to explicitly resolve mesoscale eddies; therefore, eddy parameterizations were applied to represent their effects on tracer

transport and mixing. The SSH variability in the subpolar gyre is well reproduced in comparison with satellite observations (Fig. 1c).” L392-395

L875-884: please add references for the ESMs.
We added a reference table in the supplementary file (Supplementary Table 1).

L930: did you use different reference salinities for the different CMIP models, which have different mean salinities of the Arctic Ocean?
We used the mean salinity suggested from observations. This is the most reasonable choice because simulations are compared to observations and using different reference values would not support inter-model comparison and model-observation comparison.

Reviewer #4

Dear reviewer,

Thank you very much for reviewing our paper and providing very helpful comments. We revised the paper following your comments. Please see our responses in detail below.

Sea level rise and fall in Last Ice Area reorganize Arctic export to the North Atlantic
by Qiang Wang, and co-authors

The authors address an important and timely aspect of changes in Arctic sea ice and freshwater content and export in the context of anthropogenic climate change. There has been considerable interest in this subject, and there have been a number of papers published, based on modelling and/or observations. These papers are well referenced in the present manuscript.

This new study addresses a potentially important aspect that has been largely missing from previous studies: namely the effect of dynamic sea-level variability. As such, it can add to the literature and be a novel contribution that advances significantly enough to be a suitable publication in a geophysical sciences journal. However, after having read this manuscript three times, this reviewer is not convinced this to be a Nature Communications publication in its present form. I have both reviewed and co-authored several papers submitted to Nature Comms. and comparable journals such as Science Advances. I believe that this could be an excellent contribution to a more specialized journal, such as Climate Dynamics, Journal of Geophysical Research (Oceans) or perhaps one of the Nature partner journals.
I believe that it needs at least “major revisions” by re-writing to improve the significance of the results and adjust to this journal’s broad readership. Therefore, I recommend that in its present form, this submission should be returned to the authors. However, I would encourage the authors to consider to re-think and re-write before submitting a new version to Nature Comms. or one of the many excellent, more specialized journals such as those noted above.

Thank you for your recognition of the importance of the study. We revised the paper following your comments, which helped to improve the story-telling, including re-ordering the figures and sections and adding more discussions about projection uncertainties. The arrangement of figures (main figures vs supplementary figures) is also done by combining comments from all reviewers.

Comments on the content and presentation:

The authors have done a very extensive analysis, and this reviewer has confidence in the soundness of the results, especially for the recent and near future scenarios. There are however so many uncertainties in what the latter part of the 21st century with bring, because of different global warming rates due differences in climate sensitivity and different emissions scenarios – as well as how a future AMOC weakening or "collapse" would affect the sea ice and freshwater in the Arctic.
Uncertainty in future projects consists of three components: model uncertainty, internal variability, and scenario uncertainty (Hawkins 2009; Hawkins 2011). We used a high resolution model with improved performance to reduce the first one, and used multi-model-mean of CMIP6 models to average out variability to focus on long-term change. However, we did miss the measure for the third component.

During revision, we added results from FESOM2 simulations under the SSP245 scenario, allowing us to discuss scenario uncertainty. The results further support our findings that Fram Strait export will become predominant under climate warming and a reversal of the export partitioning is expected to occur only under extremely warming conditions. Therefore, comparing the two scenarios not only informs the scenario uncertainty of future projections, but also further confirms the identified mechanism responsible for changes in Arctic exports. We added the following text and figures:

"Global warming is projected to reach approximately 3 K above the pre-industrial level by the mid-21st century under the CMIP6 SSP585 scenario (Supplementary Fig. 6). The analysis above shows that the reversal in the SSH trend in the eastern LIA is projected to occur beyond this threshold (Figs. 5a and 6a). Under the CMIP6 SSP245 scenario, global warming is expected to remain below 3 K (Supplementary Fig. 6). Accordingly, both Fram Strait export and SSH in the eastern LIA are projected to increase without exhibiting a clear reversal (Supplementary Fig. 7). This contrast indicates that the projected trend reversal in Fram Strait export is sensitive to emission pathways and may not occur under moderate warming scenarios. At the same time, the consistent increase in Fram Strait export across scenarios highlights the robustness of the projection that it is likely to become increasingly dominant in the coming decades." (Lines 225-235)

"In this study, we primarily focus on the CMIP6 SSP585 scenario, including the high-resolution simulations described above and the coarse-resolution CMIP6 models described below. SSP585 represents a high-emission pathway characterized by continued fossil fuel-driven development \cite{ONeill2016}, leading to pronounced warming by the end of the 21st century (Supplementary Fig. 6). To assess the robustness of our main findings and their sensitivity to different warming pathways, we

also employed a high-resolution FESOM2 simulation under the SSP245 scenario. SSP245 represents an intermediate pathway with moderate mitigation efforts, resulting in lower global warming compared to SSP585 (Supplementary Fig. 6). This simulation is the same as the reference simulation described above, except for the forcing scenario. All future projection results presented in this paper are based on the SSP585 scenario, except for Supplementary Fig. 7, which shows results from the SSP245 simulation." (Lines 378-389)

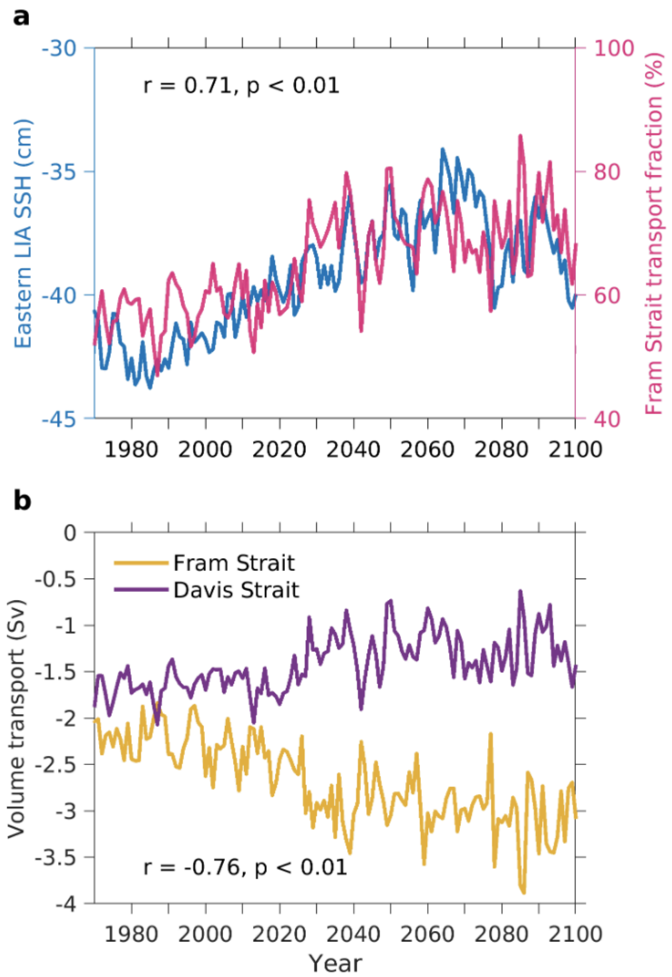

Supplementary Fig. 7: Changes in Arctic exports and sea surface height (SSH) under the SSP245 scenario. (**a**) SSH in the eastern Last Ice Area (LIA) versus the fraction of Fram Strait volume transport relative to total Arctic export (the sum of Fram and Davis straits volume transports) in the high-resolution simulation. (**b**) Volume transports through Fram and Davis straits. Negative values indicate export from the Arctic. Correlation coefficients between the two variables in each panel are shown. Under the SSP245 scenario, the Fram Strait export is projected to increase, without a clear reversal in the 21st century.

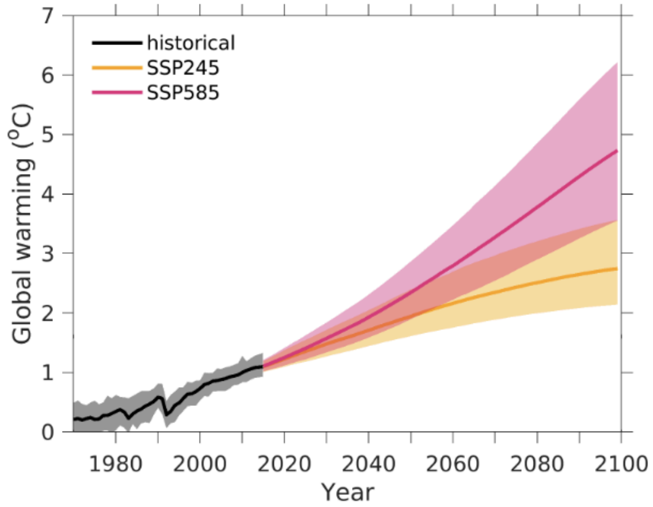

Supplementary Fig. 6: Global warming relative to the pre-industrial level under different scenarios. The solid lines show CMIP6 multi-model mean, and shadings indicate the 5th-95th percentile range (Lee et al., 2021)

Ref:
Hawkins, E., and R. Sutton, 2009: The Potential to Narrow Uncertainty in Regional Climate Predictions. *Bull. Amer. Meteor. Soc.*, **90**, 1095–1108, https://doi.org/10.1175/2009BAMS2607.1.
Hawkins, E., Sutton, R. The potential to narrow uncertainty in projections of regional precipitation change. *Clim Dyn* **37**, 407–418 (2011). https://doi.org/10.1007/s00382-010-0810-6

In my opinion, the order of the presentation could be adjusted, as follows After briefly introducing the hypothesis and the suggestions from the modelling projections, which will be presented in detail afterwards, first present the observational variability and trends (and the ability of the model to simulate this, i.e., sometime after lines 105-112, and show Figure 5c,d as the new Figure 1a,b. Then follow this with the panels in Fig5a,b and e,f – i.e., show these six panels in the new Figure 1
We re-order the result sections. Now we first show past changes and show projection results afterwards. Fig. 1 contains the six panels from original Fig. 5, and Fig. 2 contains the remaining three panels from original Fig. 5. In the Introduction section, we do not refer to figures. In the first result section, we refer to Figs. 1 and 2. The order of panels inside Fig. 1 is mainly determined by the ease of telling the story.

The figures are informative, both spatially and temporally, and generally well done, with further results relegated to the Supplementary materials. Figure 2 is of central

importance, and the consequences of circulation changes are well illustrated spatially. For example, Figure 6a and especially 6b are amongst the most interesting results that of most importance for the downstream impacts in the North Atlantic, (although these impacts are not well developed in the paper). I think that however some of the multi-panel figures are too “busy”, particularly Figure 5.
As replied above, we split Fig. 5 to new Fig. 1 and Fig. 2 now.

Furthermore, during the revision, we added Pacific Water tracer simulations to better illustrate how the discussed physical changes may influence the spreading of biogeochemically relevant matter. Here the Pacific Water is taken as an example due to its special property. We hope that the implications of the physical changes are better illustrated, although more dedicated studies are warranted in the future; we believe that our findings will inspire more studies in this research direction.

“The contrasting spatial distributions of the two tracers also imply potential regional ecosystem impacts, because Arctic waters differ markedly from North Atlantic waters in nutrient content and carbonate chemistry [12, 56]. Pacific Water, for instance, is characterized by relatively low salinity and high nutrient concentrations [57, 58]. It is further modified during its transit through the Arctic (e.g., enhanced acidification and mixing with riverine dissolved organic carbon) [56, 59]. To track its fate, we released an additional dye tracer at Bering Strait. In the historical period, Pacific Water is mainly exported through the CAA, and its spreading closely resembles that of the CAA tracer (Supplementary Fig. 9a). By the mid century, however, its export is increasingly redirected toward the Fram Strait, leading to a spreading pattern that more closely resembles that of the Fram Strait tracer (Supplementary Fig. 9b). These results highlight how shifts in Arctic export pathways can alter the delivery of biogeochemically distinct water masses to the subpolar North Atlantic. The consequences for regional ecosystems warrant dedicated future study.” (Lines 283-296)

“To illustrate the impacts of changes in Arctic export partitioning on biogeochemically relevant matter in the subpolar North Atlantic, we introduced a dye tracer restored to unity at Bering Strait to track the fate of Pacific Water. The tracer was released for 14 years starting in 1980 and 2050, respectively. In combination with the Fram Strait and Davis Strait tracers described above, these experiments illustrate how climate change is expected to reorganize the distribution of Pacific-origin water in the subpolar North Atlantic.” (Lines 358-364)

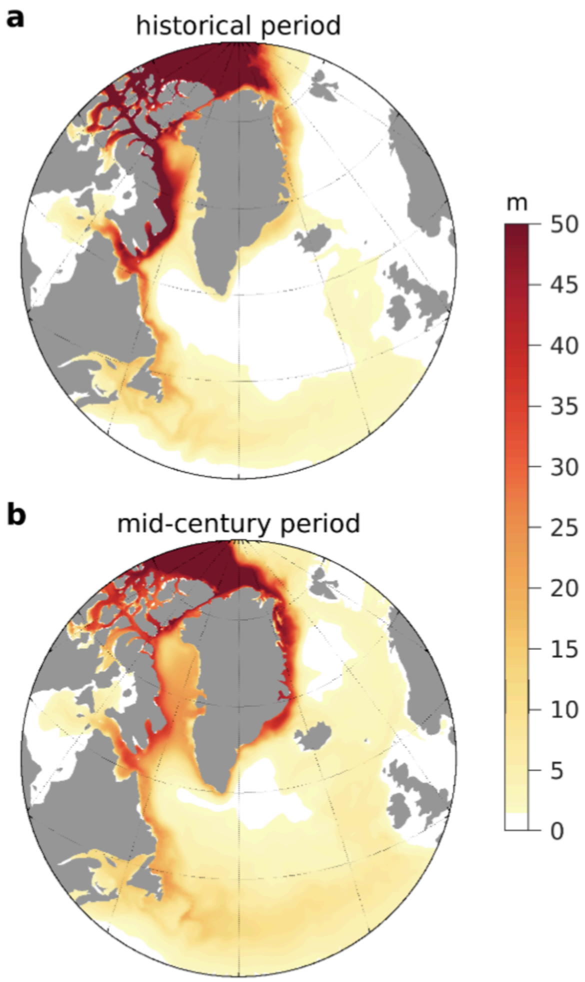

Supplementary Fig. 9: Future changes in the spatial distribution of Pacific Water. **(a,b)** Pacific Water inventory during the (a) historical and (b) mid-century periods. A dye tracer equal to one was release in the Bering Strait starting in 1980 and 2050, respectively. Shown is the vertically integrated tracer concentration, representing Pacific Water inventory, averaged over 1994 and 2064 (that is, 14 years after the start of the release). In the historical period, the spreading of Pacific Water closely resembles that of the Canadian Arctic Archipelago (CAA) tracer shown in Fig. 8b, whereas in the mid-century period it more closely resembles the Fram Strait tracer shown in Fig. 8c.

Regarding the section “Changing LIA dynamic sea level”: Is the sea ice in the LIA important in this context? That is, does the evolution of the sea ice in this region matter, or it really the broader Beaufort Gyre and Arctic Ocean that controls the circulation in the region?

The dynamic sea level in the eastern LIA determines the orientation of the flow at the end of the Transpolar Drift as we presented. And it is the overall sea ice decline and wind changes that drive the Arctic circulation changes, which is further explained in the

mechanism section later. To avoid confusion, we changed the section title to “Future changes in dynamic sea level and Arctic circulation”.

Regarding the conclusion: “The recently emerging sea level rise in the LIA indicates that the Arctic-Atlantic linkage could already be tracking the projected trajectory toward a regime dominated by Fram Strait export.” The freshwater export (solid and liquid – same ingredients, different mix) has been long established to be dominated by Fram Strait, so what is the significance of these new results? If it is the liquid freshwater transported through Fram Strait that is becoming dominant versus the CAA, then this should be stated clearly.
We added “liquid freshwater” in the summary to make it clear that oceanic transport is studied. “...with Arctic liquid freshwater export becoming dominant through Fram Strait.” (L305)

And you raised a very good point to discuss at the end:
“The Arctic supplies freshwater to the subpolar North Atlantic partly through sea ice export, primarily via Fram Strait. However, ongoing Arctic sea ice decline has led to a substantial reduction in this export pathway [60, 61]. As a result, oceanic liquid freshwater fluxes are becoming the dominant Arctic freshwater source to the subpolar North Atlantic [52]. In this context, the mechanisms driving the reorganization of liquid freshwater export identified in this study are expected to become increasingly important. At the same time, the continued loss of multiyear sea ice in the LIA [37,62] is also making in situ and satellite observations more feasible, thereby enabling improved monitoring of this climatically important region.” (L307-315)

Presentation quality:

The manuscript is thoroughly referenced, and writing quality is adequate throughout. In addition to telling the story better for a broader readership, there are several minor writing improvements needed for further clarity. For example:
Title: It should say “…Arctic Ocean freshwater export...”
We revised the paper title as you suggested.

Abstract (and elsewhere): It should be made immediately clear that these are modelling outcomes. “Here we show that….” can be rephrased as “Here we present modelling results that show that….”
We revised the abstract: “Here we use high-resolution simulations and CMIP6 data to show that …”

We also make it clearer that results are from model simulations. For example, “In this study, using high-resolution model simulations, we find that …” (L81); “To assess how dynamic sea level and Arctic export may evolve in the future, we employ both dedicated high-resolution simulations and CMIP6 models in the following sections.” (Lines 114-116)

Reviewer #1

Dear reviewer,

Thank you very much for helping improve the manuscript. We revised the paper following your comments.

The authors addressed the major concerns raised in the initial review through substantial revisions to both the analysis and presentation of the manuscript. In particular, they strengthened the mechanistic interpretation linking dynamic sea level changes in the eastern Last Ice Area to Arctic export redistribution by adding new circulation diagnostics, clarified methodological aspects related to model resolution and gateway transports, and improved the organization and terminology throughout the paper. Additional validation, sensitivity analyses, and new supplementary figures were also included to better support the robustness of the results and their broader climatic implications. Overall, the revised manuscript is significantly improved in clarity, rigor, and presentation.

The remaining vulnerabilities are primarily related to the strength of the causal interpretation relative to the analyses presented. The manuscript sometimes uses very strong wording such as “governs,” “determines,” or “dominates,” even though the conclusions are mainly supported by correlations, regression analyses, and sensitivity experiments rather than a full dynamical attribution framework. While the proposed mechanism is scientifically convincing and well supported, some statements may imply a stronger level of causal proof than has been formally demonstrated. To strengthen the manuscript before publication, the authors could slightly soften some of the causal language (e.g., replacing “governs” with “strongly influences” or “is a primary control on”), which would make the interpretation more balanced and more robust to reviewer or editorial scrutiny without weakening the scientific impact of the study.
Thanks for the suggestion. We softened these expressions. See our replies below.

Minor comments

Line 82: first mention of “eastern LIA” without proper geographic reference. Add “(Fig. 2a)”.
We change it to "north of Greenland" here, for which we do not need to refer to Fig. 2 in the introduction section. (Line80)

Line 95: define FWC (freshwater content) here. No need to do it multiple times throughout the manuscript. The authors alternate quickly between full term and acronym in adjacent sentences. Not wrong, but slightly repetitive in some sections.
To reduce the number of used acronyms, we now avoid using FWC in the main text. It is only used in figure axis labels due to space limits, and thus redefined in figure captions according to editorial requirement.

Line 135: be consistent with hyphenation, use “mid-century” or “mid century”, not both.
We keep the hythen. (L134)

Line 168–169: wording may be slightly too strong: “This evolution determines…” and “major control on…” could be softened slightly to better match the evidence presented.
The expressions are changed to "strongly influences" and "primary control" (L167-168)

Line 190: “The regression coefficient… is about 70% larger…” This is okay mathematically, but stylistically could be: “approximately 70% larger”
Suggestion taken. (L189)

Line 191: “exerts the dominant influence” may sound stronger than the regression analysis alone can fully demonstrate.
We change the phrase to "plays a more prominent role" (L190)

Line 202: “David Strait”, should be “Davis Strait”
Corrected. (L201)

Line 298–300: “key region governing…” and “largely determines…” may again benefit from slightly softer causal wording
The phrases are changed to "key region influencing..." and "strongly modulates...". (L298-300)

Line 307-308: sentence reads awkwardly. A suggestion could be “The Arctic supplies freshwater to the subpolar North Atlantic (partly through sea ice export) primarily via Fram Strait.”
We revise the sentence: "The Arctic supplies freshwater to the North Atlantic through both liquid freshwater transported by ocean currents and solid freshwater exported as sea ice. Sea ice export occurs primarily through Fram Strait." (L309-311)

Line 342: “…, about 30 km wide at its narrowest section”
Corrected. (L345)

Line 433–434: “supporting a dominant dynamical role…” could potentially be softened to “supporting an important dynamical role…”.
Suggestion taken. (L439)

Fig. 6 caption: “Solid strait lines in (b-d) denote linear fits.” Should be: “Solid straight lines…”
Corrected.

Fig. 2 caption: “The plots highlight recent changes…” Suggestion: “The panels highlight…”
Suggestion taken.

Reviewer #3

Dear reviewer,

Thank you very much for helping improve the manuscript. We revised the paper following your comments.

I thank the authors for the work they have done on the MS. They have answered all my comments and I only have a one minor remaining:

I think that the examples that you bring up in “(e.g., enhanced acidification and mixing with riverine dissolved organic carbon)”, are quite specific, and may not be very representative. Could you just write "due to biogeochemical processes and mixing with other watermasses?"
We took your suggested text. (L287-288)
